# The effect of a 24-week training focused on activities of daily living, muscle strengthening, and stability in idiopathic inflammatory myopathies: a monocentric controlled study with follow-up

**DOI:** 10.1186/s13075-021-02544-5

**Published:** 2021-06-21

**Authors:** Maja Špiritović, Barbora Heřmánková, Sabína Oreská, Hana Štorkánová, Olga Růžičková, Lucia Vernerová, Martin Klein, Kateřina Kubínová, Hana Šmucrová, Adéla Rathouská, Petr Česák, Martin Komarc, Václav Bunc, Karel Pavelka, Ladislav Šenolt, Heřman Mann, Jiří Vencovský, Michal Tomčík

**Affiliations:** 1 Institute of Rheumatology, Na Slupi 4, 128 00 Prague 2, Czech Republic; 2 Department of Physiotherapy, Faculty of Physical Education and Sport, Charles University, Prague, Czech Republic; 3 Department of Rheumatology, First Faculty of Medicine, Charles University, Prague, Czech Republic; 4 Department of Human Movement Laboratory, Faculty of Physical Education and Sport, Charles University, Prague, Czech Republic; 5 Department of Methodology, Faculty of Physical Education and Sport, Charles University, Prague, Czech Republic

**Keywords:** Myositis, Activities of daily living, Resistance training, Stability, Strength, Endurance

## Abstract

**Background:**

The structural and functional changes of the skeletal muscles in idiopathic inflammatory myopathies (IIM) caused by inflammation and immune changes can be severely disabling. The objective of this study was to assess the effect of a 24-week program combining a supervised training of activities of daily living (ADL), resistance, and stability with home exercise for improving muscle function, compared to a daily home-based exercise representing the regular outpatient care.

**Methods:**

Fifty-seven patients with IIM were consecutively and non-selectively enrolled in an intervention (IG, *n* = 30) or control (CG, *n* = 27) group. Both groups were provided a standard-of-care pharmacological treatment and follow-up. Only the IG underwent the supervised intervention twice a week for 1 h per session. At baseline, 12, 24, and 48 weeks, all patients were assessed by an assessor blinded to the intervention for primary outcomes: muscle strength (Manual Muscle Testing of eight muscle groups [MMT-8]) and endurance (Functional Index-2 [FI-2]), and secondary outcomes: stability and body composition. Secondary outcomes also included questionnaires evaluating disability (Health Assessment Questionnaire [HAQ]), quality of life (Short Form 36 [SF-36]), depression (Beck’s Depression Inventory-II [BDI-II]), and fatigue (Fatigue Impact Scale [FIS]), and analysis of the systemic and local inflammatory response and perceived exertion to assess the safety of the intervention.

**Results:**

Twenty-seven patients in the IG and 23 in the CG completed the entire program and follow-up. At week 24, compared to deterioration in the CG, we found a significant improvement in the IG in muscle strength (mean % improvement compared to baseline by 26%), endurance (135%), disability (39%), depression (26%), stability (11%), and basal metabolism (2%) and a stabilization of fitness for physical exercise. The improvement was clinically meaningful (a 24-week change by >20%) in most outcomes in a substantial proportion of patients. Although the improvement was still present at 48 weeks, the effect was not sustained during follow-up. No significant increase in the systemic or local expression of inflammatory markers was found throughout the intervention.

**Conclusions:**

This 24-week supervised intervention focused on ADL training proved to be safe and effective. It not only prevented the progressive deterioration, but also resulted in a significant improvement in muscle strength, endurance, stability, and disability, which was clinically meaningful in a substantial proportion of patients.

**Trial registration:**

ISRCTN35925199 (retrospectively registered on 22 May 2020).

**Supplementary Information:**

The online version contains supplementary material available at 10.1186/s13075-021-02544-5.

## Background

Idiopathic inflammatory myopathies (IIM, briefly myositis) are a heterogeneous group of rare disorders characterized by chronic inflammation of skeletal muscles and multiple organ involvement associated with muscle weakness and low muscle endurance [1]. IIM are associated with significant morbidity and mortality if not properly managed during the early stages of the disease [2]. The widely recognized subsets of IIM are dermatomyositis (DM), polymyositis (PM), and inclusion body myositis (IBM). Recently, other subsets of IIM have been defined such as clinically amyopathic dermatomyositis (CADM), antisynthetase syndrome (ASS), and immune-mediated necrotizing myopathy (IMNM) [3]. Recent findings suggest that both immune and nonimmune mechanisms are involved in the pathogenesis of myositis and that different molecular pathways might predominate in different subsets of myositis [4]. Although the new insights into pathogenesis are paving the way for improvements in early diagnosis, classification, and treatment, IIM remain a major medical challenge [4, 5].

Muscle weakness is a characteristic feature of IIM and is mostly localized symmetrically to the proximal muscle groups of the limbs, torso, and neck. However, in patients with IBM, muscle weakness tends to be more asymmetric and more often affects the distal muscles [6]. Muscle weakness in IIM is usually not accompanied by myalgia, but if so, the pain may appear in an acute condition, e.g., in IMNM or ASS [3]. Furthermore, muscle atrophies develop gradually. Thus, during the course of the disease, it is essential to distinguish between the various causes of muscle symptoms, such as persistent active inflammation and the subsequent damage or atrophy [3]. IIM are not merely muscle disorders, as other organs are often affected, such as the skin in DM and the lungs, heart, joints, and gastrointestinal tract in both PM and DM, suggesting that these are systemic autoimmune diseases [1]. Muscle weakness or muscle atrophy is highly disabling and could hinder activities of daily living (ADL). Impaired muscle function and consequent fatigue can lead to reactive depression and have a significant impact on the capacity for work, which renders an additional economic burden on health care and society, as IIM have the highest incidence during the patients’ highly productive decade of working life [7, 8].

The main goal of the treatment of IIM is to improve muscle strength, suppress the extramuscular activity of the disease, and at the same time, minimize the risk of treatment-related complications [3]. While inflammation can be attenuated by immunosuppressive therapy with subsequently improved muscle strength, muscle damage can only be ameliorated by exercise and targeted physiotherapy. This represents a window of opportunity for non-pharmacological interventions, in both prevention of further progression and reduction of already established disability and impaired quality of life [5]. However, regular physical therapy is often unavailable to the patients, due to the lack of experienced physiotherapists with experience and specific skills required for treating IIM patients. Although it has long been feared that physical exercise exacerbates muscle inflammation and consequently aggravates the clinical manifestations of IIM, over the past years, rehabilitative treatment of IIM has gained increasing attention and demonstrated to be safe and effective to a varying extent [9, 10]. However, only a limited number of studies on the subject are published. These studies assess the effect of a broad range of treatment modalities and their combinations with various outcomes and are often limited by a small number of patients, short period of intervention, and lack of a control group, follow-up, or a blinded assessor [9, 10]. To our knowledge, five systematic reviews provide a comprehensive overview of the already existing evidence on the effect and safety of non-pharmacological interventions in IIM and analyze the quality and limitations of the published studies [11–15]. However, the optimal training intervention for IIM patients has yet to be established. Thus, there is an unmet need to provide further evidence on efficacy and safety supporting the implementation of non-pharmacological treatment in routine clinical practice.

Considering the lack of available data on rehabilitative treatment in IIM, we aimed to demonstrate the efficacy of a 24-week intervention combining a supervised, tailored program focused on activities of daily living, resistance, and stability training (ADLRSp), and home-based exercise, evidenced by multiple outcome measures reflecting the muscle function, disability, and quality of life, compared to the usual outpatient care with home exercise in a substantial number of IIM patients.

## Methods

### Study design

This prospective, controlled, assessor-blinded, non-randomized, single-center study (ISRCTN35925199) with follow-up was conducted from May 2014 to June 2017. Approval was obtained from the Ethics Committee of the Institute of Rheumatology in Prague (IoRP) prior to initiation of the study. Patients’ written informed consent was obtained at enrollment. Patients fulfilling inclusion criteria were consecutively and non-selectively enrolled and were allocated to an intervention (IG) or control group (CG) based on their ability to adhere to the protocol of the supervised ADLRSp. At enrolment, all patients were educated on general preventive measures and received educational materials for daily ADL home exercise, which represents the usual standard of care. Only the IG underwent the 24-week supervised ADLRSp, twice weekly for 1 h per session, with a subsequent 24-week follow-up period. CG patients during weeks 0–48 and IG patients during weeks 24–48 were asked to refrain from starting any regular hospital-based non-pharmacological intervention.

### Inclusion criteria

All patients (≥ 18 years of age) had to fulfill the Bohan/Peter criteria for DM/PM [16, 17] or ENMC criteria for IMNM [18] or IBM [19]. The patients had to be regularly followed by their attending rheumatologist, treated with a standard-of-care pharmacotherapy, and stable for ≥ 1 month prior to inclusion and have a decreased muscle strength defined as (a) a total score of ≤ 64/80 in Manual Muscle Testing of 8 muscle groups (MMT-8ts) [20] and/or (b) individual MMT score of ≤ 7/10 in ≥ 1 proximal muscle group.

### Exclusion criteria

Exclusion criteria comprised any other condition, including medical (e.g., active neoplasia, severe lung or heart conditions and comorbidities contraindicating exercise) or incompatible psychiatric disorders, participation in a clinical trial in the previous 3 months, and inclusion in a standardized physical therapy program in the previous 6 months.

### Outcome measures

Primary outcome measures included MMT-8 [20] and Functional Index-2 (FI-2) [20]. Secondary outcome measures included the Health Assessment Questionnaire (HAQ) [20], Medical Outcomes Study 36-item Short Form Health Survey (SF-36) [20, 21], Fatigue Impact Scale (FIS) [22], Beck’s Depression Inventory-II (BDI-II) [23], force vector area (FVA) assessed by indirect static posturography [24], basal metabolic rate (BMR) [25], and muscle fitness (extracellular mass to body cell mass ratio [ECM/BCM]) assessed by bioelectric impedance [26, 27]. Safety was assessed by (a) Borg Category-Ratio (CR)-10 perceived exertion scale [28, 29]; (b) erythrocyte sedimentation rate (ESR), serum C-reactive protein (CRP), creatine kinase (CK), lactate dehydrogenase (LD), myoglobin, and selected inflammatory cytokines (interleukin [IL]-1β, IL-6, tumor necrosis factor [TNF]) or chemokines (IL-8, monocyte chemoattractant protein-1 [MCP-1, CCL2]) which play a substantial role in the pathophysiology of IIM [1, 3, 30–34]; and (c) mRNA expression of these cytokines/chemokines assessed in the muscle biopsy of volunteers from the IG (*n* = 7) at week 0 and 24. Finally, the three available core set outcome measures (MMT-8, HAQ, and CK) were used to assess clinical response in accordance with the 2016 American College of Rheumatology (ACR)/European League Against Rheumatism (EULAR) criteria [35].

At baseline, and weeks 12, 24, and 48, objective outcomes were evaluated by an assessor blinded to the intervention, and questionnaires were self-administered and returned in a sealed envelope to the blinded assessor.

### Intervention program

Each supervised session of the ADLRSp started with a warm-up (10 min) and concluded with stretching and breathing relaxation (10 min). The first day of the week focused on the training of activities of daily living (40 min), which included elements from developmental kinesiology, stork walk, marching, handling of loads, hanging clothes, combing, putting on brassieres, and isometric strengthening of hip joint adductors. The second day of the week focused on resistance training (30 min) and stability training (10 min).

### Statistical analysis

Basic descriptive statistics (mean, median, standard error of the mean [SEM], inter-quartile range [IQR], skewness, and kurtosis) were computed for all variables, which were subsequently tested for normality using the Kolmogorov–Smirnov and Shapiro–Wilk tests. Baseline differences between IG and CG in selected parameters were analyzed using the Mann–Whitney U test (for continuous variables) and the chi-square test (for categorical variables). Two-way repeated measures ANOVA (interaction: group × time) was used to compare parameter changes over time between the IG and CG. A significant interaction was then followed by one-way repeated measures ANOVA within each group, using Fisher’s least significant difference (LSD) post hoc comparisons. The ANOVA tests were also adjusted for covariates, which were significantly different between the IG and CG at baseline (i.e., MMT-8ts, the current prednisone equivalent dose [CPED], and CK).

Inspired by the IMACS preliminary definitions of improvement using the core set measures [36], an absolute percentage change over weeks 0–24 was calculated, according to which the patients were stratified into five categories: (a) improved by >20%, which is considered clinically significant; (b) improved by ≤20% and >0%; (c) unchanged; (d) deteriorated by ≤20% and >0%; and (e) deteriorated by >20%. However, for FVA, BMR, and ECM/BCM, in which the most considerable change was observed at week 12, an absolute percentage change over weeks 0–12 was calculated. The difference in the distribution of IG and CG in these five categories, as well as in the categories according to the 2016 ACR/EULAR response criteria [35], was tested using the chi-square test and was visualized using a tree diagram.

Data are presented as median (IQR) unless stated otherwise. Statistical significance was set at *p* < 0.05. All analyses were conducted using SPSS version 25 (SPSS, Inc., Chicago, IL, USA). Graphs were created using GraphPad Prism (version 6; GraphPad Software, La Jolla, CA, USA).

Further details on the outcome measures, intervention program, safety and adherence monitoring, and clinical and laboratory assessments are available in the Supplementary Material.

## Results

### Patient inclusion and characteristics

**Fig. 1:**
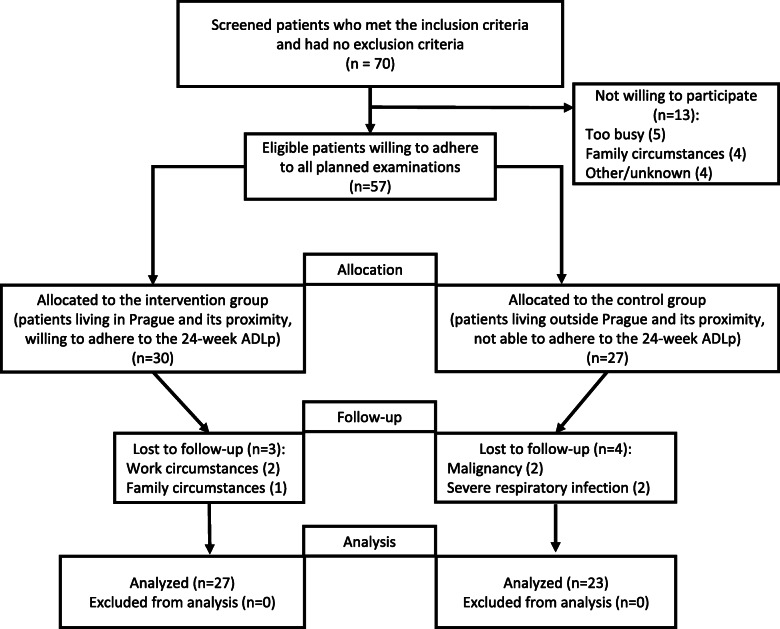
Flow diagram of the training of activities of daily living, muscle strengthening, and stability in idiopathic inflammatory myopathies

Out of 70 IIM patients, who met the inclusion criteria and lacked the exclusion criteria according to their medical records, 57 were willing to adhere to all planned examinations (Fig. 1). These patients were allocated into the IG (mostly patients living in Prague and its vicinity, *n* = 30) or the CG (mostly patients living further away, *n* = 27) based on their motivation to undergo the intervention and ability to adhere to the study protocol. A total of 27/30 patients in the IG (90%) and 23/27 patients in the CG (85%) completed the study (*p* = 0.6968). Reasons for drop-outs included family or work circumstances in the IG and severe respiratory infection and malignancy in the CG.

The baseline demographic and disease characteristics of all patients included in the analysis are shown in Table 1. Despite the lack of randomization, the only statistically significant differences between the groups were found in three parameters: patients in the CG had a higher MMT-8ts, lower serum CK levels, and a higher current prednisone equivalent dose. These differences were consistent with a numerical trend towards shorter disease duration and lower serum myoglobin in the CG, which did not reach statistical significance (Table 1). Thus, only MMT-8ts, CK, and CPED were adjusted for in the statistical analysis.

**Table 1:** Clinical and demographic characteristics of myositis patients analyzed in the intervention group and control group

Parameters	Intervention group (*n* = 27)	Control group (*n* = 23)	*p*-value
Gender: female/male, n (%)	22 (81)/5 (19)	21 (91)/2 (9)	0.429
Age, years	56.0 (41.0–68.0)	58.0 (52.0–65.0)	0.513
**Clinical features**			
Disease duration, years	6.0 (2.0–9.4)	2.8 (1.4–8.3)	0.266
IIM subtype, n (%): PM/DM/IMNM	12 (44)/10 (37)/5 (19)	10 (44)/11 (48)/2 (8)	0.064
IIM-associated symptoms, n (%):			
MW/D/SR/MH/	27 (100)/5 (19)/2 (7)/1 (4)/	23 (100)/7 (30)/4 (17)/3 (13)/	1.000/0.319/0.379/0.306/
RP/A/ILD/CI	3 (11)//1 (4)/9 (33)/4 (15)	7 (30)//1 (4)/7 (30)/5 (22)	0.081/1.000/1.000/0.477
MMT-8 total score	56.0 (48.0–65.0)	64.0 (55.0–71.0)	**0.024**
MITAX	0.2 (0.1–0.3)	0.2 (0.1–0.4)	0.120
MDI extent	0.1 (0.0–0.1)	0.1 (0.0–0.1)	0.355
**Laboratory features**			
Autoantibodies, n (%):			
ANA/Mi-2/TIF1/MDA5/	17 (63)/2 (7)/0 (0)/0 (0)/	14 (61)/1 (4)/2 (9)/1 (4)/	1.000/1.000/0.186/0.437/
SAE/NXP2/SRP/HMGCR/Jo-1/	0 (0)/2 (7)/1 (4)/3 (11)/3 (11)/	1 (4)/1 (4)/2 (8)/0 (0)/7 (30)/	0.437/1.000/0.574/1.000/0.081/
PM-Scl/snRNP/Ku/Ro	2 (7)/2 (7)/2 (7)/11 (41)	2 (9)/0 (0)/0 (0)/8 (35)	1.000/1.000/1.000/0.497
CRP, mg/L	3.0 (1.7–4.6)	4.1 (1.4–8.2)	0.403
ESR, mm/h	13.0 (7.0–26.0)	15.0 (9.0–30.0)	0.550
CK, μkat/L	4.0 (1.8–8.9)	1.3 (0.8–3.9)	**0.005**
LD, μkat/L	4.0 (3.5–4.9)	3.7 (3.4–4.7)	0.626
Myoglobin, μg/L	112.3 (67.0–299.5)	78.0 (47.6–128.2)	0.108
**Current treatment**			
Prednisone equivalent dose, mg/day	6.0 (1.3–10.0)	15.0 (5.0–30.0)	**0.021**
MTX/CPA/AZA/CSA/	10 (37)/0 (0)/7 (26)/4 (15)/	7 (30)/1 (4)/2 (9)/2 (9)/	0.767/0.460/0.152/0.674/
LEF/MMF/SAS/HQ/TAC/	0 (0)/2 (7)/1 (4)/0 (0)/1 (4)/	2 (9)/0 (0)/0 (0)/1 (4)/0 (0)/	0.207/0.493/1.000/1.000/1.000/
RTX/IVIg, n (%)	0 (0)/0 (0)	0 (0)/0 (0)	1.000/1.000

Acronyms: Data are presented as median (inter-quartile range) unless stated otherwise. Statistically significant differences (*p* < 0.05) are marked in bold

*IIM* idiopathic inflammatory myopathy, *PM* polymyositis, *DM* dermatomyositis, *IMNM* immune-mediated necrotizing myopathy, *MW* muscle weakness, *D* dysphagia, *SR* skin rash, *MH* mechanic’s hands, *RP* Raynaud’s phenomenon, *A* arthritis, *ILD* interstitial lung disease, *CI* cardiac involvement, *MMT-8* Manual Muscle Testing of eight muscles, *MITAX* Myositis Intention to Treat Activity Index, *MDI* Myositis Damage Index, *ANA* antinuclear antibodies, *Mi-2* antinuclear helicase 218/240 kDa, *TIF1* anti-TIF1 (transcription intermediary factor-1), *MDA5* anti-CADM-140 (melanoma differentiation-associated gene 5), *SAE* anti-SUMO1 (small ubiquitin-like modifier 1) activating enzyme, *NXP2* anti-NXP2 (nuclear matrix protein), *SRP* anti-signal recognition particles, *HMGCR* anti-3-hydroxy-3-methylglutaryl-CoA reductase, *Jo-1* anti-histidyl-tRNA synthetase, *PM-Scl* anti-Pm-Scl (anti-core complex 11-16 proteins), *snRNP* small nuclear ribonucleoprotein, *Ku* anti-Ku (against the nuclear DNA-dependent protein kinase subunit), *Ro* anti-Ro (52/60 kDa, against cytoplasmic RNA and associated peptides), *CRP* C-reactive protein, *ESR* erythrocyte sedimentation rate, *CK* creatine kinase, *LD* lactate dehydrogenase, *MTX* methotrexate, *CPA* cyclophosphamide, *AZA* azathioprine, *CSA* cyclosporin A, *LEF* leflunomide, *MMF* mycophenolate mofetil, *SAS* sulphasalazine, *HQ* hydroxychloroquine, *TAC* tacrolimus, *RTX* rituximab, *IVIg* intravenous immunoglobulins

### Primary outcomes

The unadjusted inter-group analysis revealed significant differences between the IG and CG throughout the experimental period in both MMT-8ts and FI-2. The significant difference in FI-2 was confirmed after adjusting for MMT-8ts, CK, and CPED (Table S1).

**Fig. 2:**
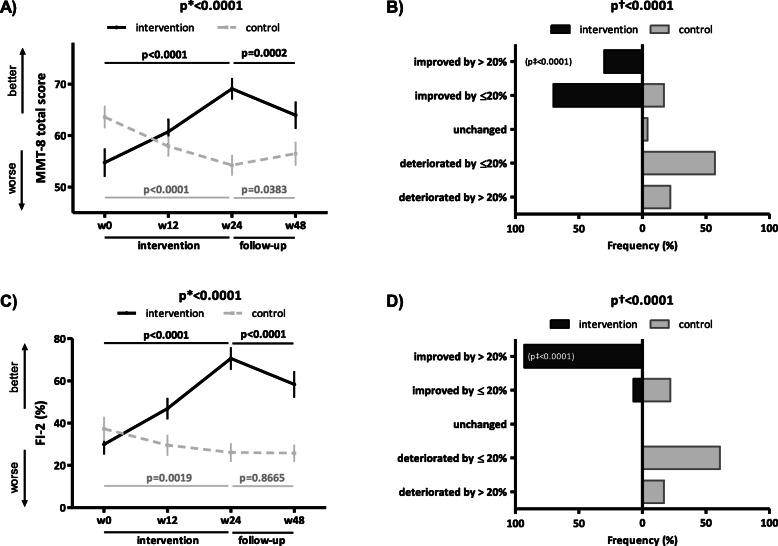
Primary outcomes objectively assessing muscle strength and endurance in the intervention and control group. In the intervention group (IG), the 24-week intervention led to a significant improvement in muscle strength objectively assessed by Manual Muscle Testing (MMT)-8 (**A**) and muscle endurance evaluated by Functional Index (FI)-2 (**C**), thus preventing the progressive deterioration observed in the control group (CG) over weeks 0–24. Although the improvement in MMT-8 (**A**) and FI-2 (**C**) in the IG was still present at week 48, the maximum effects were not sustained. In the IG, the improvement in MMT-8 (**B**) and FI-2 (**D**) was clinically meaningful (a 24-week improvement by >20%) in a substantial proportion of patients. Data in **A** and **C** are presented as the mean ± standard error of the mean (whiskers). Data in the tree diagrams (**B** and **D**) present the percentage distribution of patients by outcome, which is stratified into five levels of improvement/deterioration over weeks 0–24. w, week; p*, unadjusted inter-group comparison by two-way ANOVA; p, unadjusted intra-group comparison by one-way ANOVA in black (IG) and gray (CG); p†, difference in the overall distribution in five levels of the patient outcome by chi-square test; p‡, difference in the category of clinically meaningful improvement by chi-square test

The unadjusted intra-group analysis of MMT-8ts and FI-2 in the IG showed a significant improvement over the first 12 weeks, the last 12 weeks, and over the entire 24-week intervention (Fig. 2A, C, Table S1), whereas in the CG, we found a significant progressive deterioration over weeks 0–24 (Fig. 2A, C, Table S1). Of particular interest, the improvement in the IG over weeks 0–24 was clinically meaningful (i.e., by > 20%) in a substantial proportion of patients (MMT-8ts 30%, FI-2 93%), which was significantly more considerable than in the CG (MMT-8ts 0%, FI-2 0%) (Fig. 2B, D). However, the maximum effect in the IG was not sustained at week 48 (Fig. 2A, C, Table S1). The adjusted intra-group analysis of FI-2 confirmed these results (Table S1). Furthermore, the detailed analysis of individual muscle groups assessed by MMT revealed a significant improvement in the IG over weeks 0–24 in virtually all ten assessed muscle groups, both in the unadjusted and adjusted analysis (with the exception of m. quadriceps femoris, possibly due to high baseline values and a ceiling effect) (Table S2).

### Secondary outcomes

The unadjusted inter-group analysis demonstrated significant differences between the IG and CG throughout the experimental period only in the measures evaluating function/disability (HAQ), depression (BDI-II), stability (FVA), basal metabolism (BMR), and muscle fitness (ECM/BCM) (Table S1). The adjusted inter-group analysis confirmed these results for all parameters except for BMR (*p* = 0.0539) and ECM/BCM (*p* = 0.1032) (Table S1). However, neither of the inter-group analyses detected any significant differences in the measures of quality of life (SF-36 PCS and MCS) and fatigue (FIS) (p > 0.05 for all) (Table S2).

**Fig. 3:**
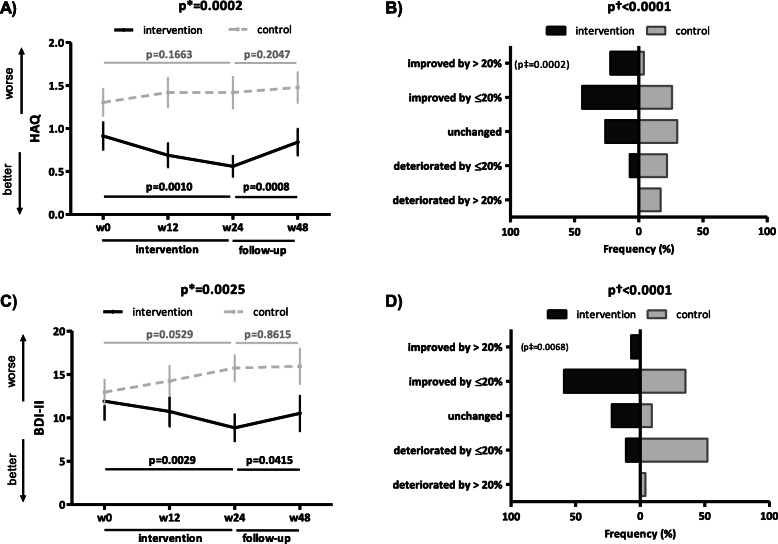
Secondary outcomes subjectively assessing function/disability and depression in the intervention and control group. In the intervention group (IG), the 24-week intervention led to a significant improvement in global function/disability subjectively assessed by the Health Assessment Questionnaire (HAQ) (**A**) and depression evaluated by Beck’s depression inventory (BDI)-II (**C**), thus preventing the trend to progressive deterioration observed in the control group (CG) over weeks 0–24. Although the improvement in HAQ (**A**) and BDI-II (**C**) in the IG was still present at week 48, the maximum effects were not sustained. In the IG, the improvement in HAQ (**B**) was clinically meaningful (a 24-week improvement by >20%) in a substantial proportion of patients. The difference in the percentage distribution of patients according to five levels of improvement/deterioration over weeks 0–24 between the IG and CG was statistically significant for BDI-II (**D**). Data in **A** and **C** are presented as the mean ± standard error of the mean (whiskers). Data in the tree diagrams (**B** and **D**) present the percentage distribution of patients by outcome, which is stratified into five levels of improvement/deterioration over weeks 0–24. w, week; p*, unadjusted inter-group comparison by two-way ANOVA; p, unadjusted intra-group comparison by one-way ANOVA in black (IG) and gray (CG); p†, difference in the overall distribution in five levels of the patient outcome by chi-square test; p‡, difference in the category of clinically meaningful improvement by chi-square test

The unadjusted intra-group analysis of HAQ and BDI-II in the IG demonstrated a significant improvement over weeks 0–24 (Fig. 3A, C), which was confirmed after adjusting (Table S1), and was clinically meaningful in a substantial proportion of patients only for HAQ (IG 22%, CG 4%, *p* = 0.0002) (Fig. 3B). Nevertheless, a significant difference in the overall distribution of outcomes between the IG and CG was found in BDI-II (Fig. 3D). However, the maximum effect in the IG was not sustained at week 48 (Fig. 3A, C, Table S1), whereas in the CG, we found a trend towards deterioration over weeks 0–24 (HAQ: *p* = 0.1663; BDI-II: *p* = 0.0529) (Fig. 3A, C, Table S1).

The unadjusted intra-group analysis of fatigue (FIS) and quality of life (SF-36) in the IG revealed a trend towards improvement in fatigue and the physical component of the quality of life (SF-36 PCS) over weeks 0–12 (*p* = 0.1098, *p* = 0.0778, respectively) and weeks 0–24 (*p* = 0.1012, *p* = 0.0567, respectively). However, the trend towards improvement in SF-36 PCS became significant after adjusting (Table S2). No significant changes were detected in the mental component of the quality of life (SF-36 MCS) in the IG (Table S2).

**Fig. 4:**
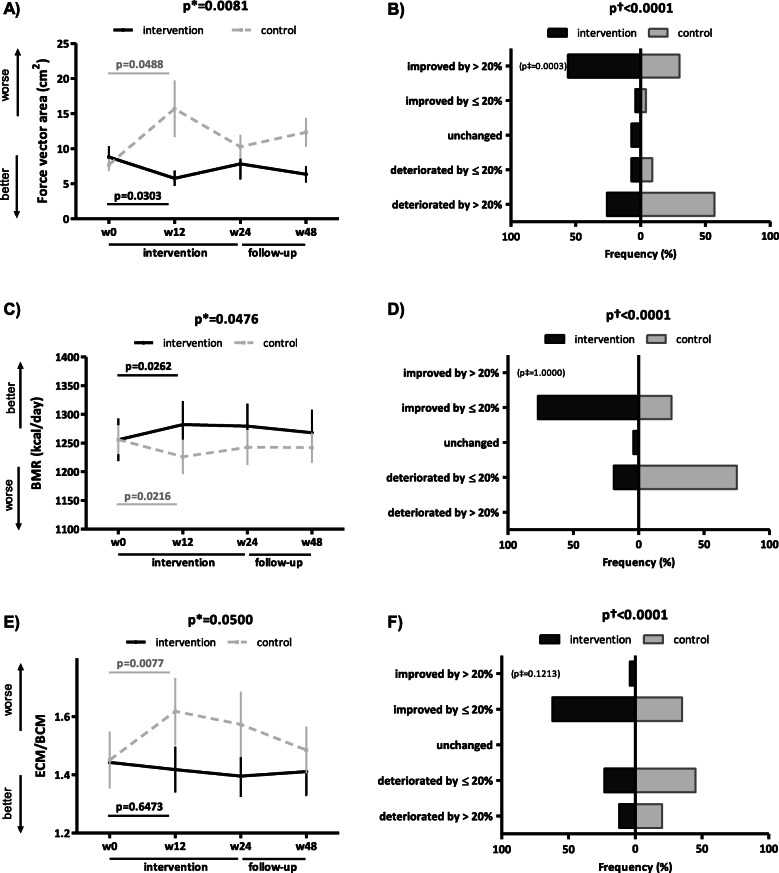
Secondary outcomes assessing stability, basal metabolism, and muscle fitness in the intervention and control group. In the intervention group (IG), the first 12 weeks of the intervention led to a significant improvement in objectively assessed stability (force vector area) (**A**), an increase in basal metabolic rate (BMR) (**C**), and stabilization of muscle fitness assessed by extracellular mass to body cell mass ratio (ECM/BCM) (**E**), thus preventing any significant deterioration observed in the control group (CG) over weeks 0–12. The improvement in stability (**A**) and BMR (**C**) and the stabilization of ECM/BCM (**E**) in the IG were still present at week 48. In the IG, the improvement in stability (**B**) was clinically meaningful (a 12-week improvement by >20%) in a substantial proportion of patients. The difference in the percentage distribution of patients according to five levels of improvement/deterioration over weeks 0–12 between the IG and CG was statistically significant for both BMR (**D**) and ECM/BCM (**F**). Data in **A**, **C**, and **E** are presented as the mean ± standard error of the mean (whiskers). Data in the tree diagrams (**B**, **D**, and **F**) present the percentage distribution of patients by outcome, which is stratified into five levels of improvement/deterioration over weeks 0–12. w, week; p*, unadjusted inter-group comparison by two-way ANOVA; p, unadjusted intra-group comparison by one-way ANOVA in black (IG) and gray (CG); p†, difference in the overall distribution in five levels of the patient outcome by chi-square test; p‡, difference in the category of clinically meaningful improvement by chi-square test

Of particular interest, most evident changes, albeit of borderline statistical significance, were apparent over weeks 0–12 in three exploratory, secondary outcomes assessing stability (FVA), basal metabolism (BMR), and muscle fitness (ECM/BCM). During this period, stability significantly improved in the IG (p_unadjusted_ = 0.0303, p_adjusted_ = 0.0623), whereas it deteriorated in the CG (p_unadjusted_ = 0.0488, p_adjusted_ = 0.1491) (Fig. 4A, Table S1). The improvement in stability in the IG was clinically meaningful in a substantial proportion of patients (IG 56%, CG 30%, *p* = 0.0003) (Fig. 4B). Furthermore, in the CG, we detected a significant decrease in basal metabolism (p_unadjusted_ = 0.0216, p_adjusted_ = 0.0052) and in muscle fitness (p_unadjusted_ = 0.0077, p_adjusted_ = 0.0009) (Fig. 4C, E, Table S1), whereas in the IG, we found a significant increase in basal metabolism (p_unadjusted_ = 0.0262, p_adjusted_ = 0.1011) and a stabilization of muscle fitness (Fig. 4C, E, Table S1). A significant difference in the overall distribution of outcomes between the IG and CG was found in both BMR and ECM/BCM (Fig. 4D, F). The improvements in these three outcomes observed in the IG at week 12 were sustained over weeks 12–48 (Fig. 4A, C, E, Table S1).

Since our data on physician’s and patient’s global activity assessed by visual analog scales (VAS), and extramuscular disease activity, were incomplete at weeks 12, 24, and 48, we decided to only use the three core set measures (MMT-8, HAQ, and CK), which were available for all patients at all time points, to assess the efficacy of our ADLRSp according to the 2016 ACR/EULAR response criteria for adult myositis [35] (Table 2). Given this limitation, the (mean ± SEM) Total Improvement Score (TIS) in the IG and CG at weeks 12, 24, and 48 (out of possible maximum TIS of 50) was as follows: week 12 (IG 18.0 ± 1.5, CG 1.0 ± 1.4, *p* < 0.0001), week 24 (IG 26.7 ± 1.9, CG 2.4 ± 0.9, *p* < 0.0001), and week 48 (IG 19.4 ± 2.1, CG 3.9 ± 1.5, *p* < 0.0001) (Table 2). Applying the 2016 ACR/EULAR response criteria modified for the three core set measures and the maximum TIS of 50, we demonstrate a minimal improvement on a group level in the IG at both week 12 (18.0 points) and 48 (19.4 points) and a moderate improvement at week 24 (26.7 points).

**Table 2:** Distribution according to the original 2016 ACR/EULAR response criteria for improvement and their modified version

Original 2016 ACR/EULAR response criteria for adult myositis of minimal (≥20 points), moderate (≥40 points), and major (≥60 points) improvement according to the Total Improvement Score (range 0–100) based on six core set measures: physician’s global activity (range 0–20), patient’s global activity (range 0–10), extramuscular disease activity (range 0–20), MMT (range 0–32.5), HAQ (range 0–10), and enzyme levels (range 0–7.5). However, the percentage distribution of patients in each category was calculated only from MMT-8, HAQ, and CK, thus amounting to a maximum Total Improvement Score of 50					
**Interval (weeks)**	**Category of improvement (points)**	**Intervention group (% of patients)**	**Control group (% of patients)**	***p*-value for each category**	***p*-value for overall distribution**
0–12	Least improvement (0–19) or worsening	48	100	**<0.001**	**<0.001**
	Minimal improvement (20–39)	52	0	**<0.001**	
	Moderate improvement (40–59)	0	0	NA	
	Major improvement (≥60)	0	0	NA	
0–24	Least improvement (0–19) or worsening	11	100	**<0.001**	**<0.001**
	Minimal improvement (20–39)	78	0	**<0.001**	
	Moderate improvement (40–59)	11	0	**<0.001**	
	Major improvement (≥60)	0	0	NA	
0–48	Least improvement (0–19) or worsening	44	91	**<0.001**	**<0.001**
	Minimal improvement (20–39)	52	9	**<0.001**	
	Moderate improvement (40–59)	4	0	**0.046**	
	Major improvement (≥60)	0	0	NA	
Modification of the original 2016 ACR/EULAR response criteria for adult myositis with minimal (≥10 points), moderate (≥20 points), and major (≥30 points) improvement due to the maximum Total Improvement Score of 50, as a sum of only three core set measures: MMT-8, HAQ, and CK, which better characterizes the overall improvement based on these three outcome measures					
**Interval (weeks)**	**Category of improvement (points)**	**Intervention group (% of patients)**	**Control group (% of patients)**	***p*-value for each category**	***p*-value for overall distribution**
0–12	Least improvement (0–9) or worsening	4	100	**<0.001**	**<0.001**
	Minimal improvement (10–19)	44	0	**<0.001**	
	Moderate improvement (20–29)	44	0	**<0.001**	
	Major improvement (≥30)	8	0	**0.004**	
0–24	Least improvement (0–9) or worsening	0	83	**<0.001**	**<0.001**
	Minimal improvement (10–19)	15	17	0.689	
	Moderate improvement (20–29)	44	0	**<0.001**	
	Major improvement (≥30)	41	0	**<0.001**	
0–48	Least improvement (0–9) or worsening	11	78	**<0.001**	**<0.001**
	Minimal improvement (10–19)	33	13	**0.001**	
	Moderate improvement (20–29)	41	9	**<0.001**	
	Major improvement (≥30)	15	0	**<0.001**	

Acronyms: Statistically significant differences (*p* < 0.05) are marked in bold

*ACR* American College of Rheumatology, *EULAR* European League Against Rheumatism, *MMT* Manual Muscle Testing, *HAQ* Health Assessment Questionnaire, *MMT-8* MMT assessing eight muscle groups, *CK* creatine kinase *NA* not applicable

### The systemic and local inflammatory response to the intervention

Since both groups underwent concurrent standard-of-care pharmacological treatment throughout the entire study, we rather aimed to exclude the pro-inflammatory effects of the intervention than to assess its potential anti-inflammatory effects.

On the systemic level, over the 24-week intervention in the IG, we did not observe any significant increase in either routine inflammatory biomarkers (CRP and ESR), or selected pro-inflammatory cytokines (IL-1β, IL-6, and TNF) or chemokines (IL-8, and MCP-1) which play a substantial role in the pathophysiology of IIM [1, 3, 30–34] (Table S3). Interestingly, in the IG, we found a significant decrease in TNF, a trend towards a decrease in MCP-1 (*p* = 0.0796), and a trend towards an increase in IL-1β (*p* = 0.0854), whereas in the CG, we detected a significant decrease in TNF and MCP-1 and a significant increase in IL-1β. In addition, an overall numerical trend towards a decrease in CK, LD, and myoglobin, which was not statistically significant, was observed in both groups over weeks 0–24 (Table S3).

In line with these findings, we did not observe any significant increase in the local mRNA expression of IL-1β, IL-6, IL-8, and MCP-1 and found a significant decrease in the mRNA expression of TNF in the muscle tissue of seven volunteers from the IG in week 0 and 24 (Table S4).

### Adverse events and adherence to the intervention

During the 24-week intervention, 9/27 patients in the IG had the following adverse events unrelated to the intervention: urinary bladder infection (*n* = 3), nasopharyngitis (*n* = 2), aggravated reflux (*n* = 2), and diarrhea/constipation (*n* = 2). All patients tolerated the intervention well, no increase in muscle exertion was found (Table S3), and 20/27 patients occasionally reported tolerable intervention-induced muscle pain and fatigue. No signs of clinically relevant deterioration (e.g., injuries, tendonitis, arthritis) were reported, and no changes in the use of analgesics were recorded. No substantial increase in levels of pain, dyspnea, and fatigue assessed by VAS, or exertion either during supervised sessions or in the diaries monitoring the performance of home-based exercise were recorded (data not shown). All 27 patients attended all 48 supervised sessions. In 10 of these patients, 1–5 sessions needed to be rescheduled on the next possible day due to patients’ unavailability.

## Discussion

In our study, we demonstrate the efficacy of a 24-week program combining a tailored, supervised training of activities of daily living, resistance, and stability (ADLRSp) with home exercise (focused on ADL) in improving several measures of muscle function and stability and mitigating general disability and depression of IIM patients, compared to a daily home exercise program representing the regular outpatient care.

To date, studies on non-pharmacological interventions in IIM have provided evidence of efficacy for various interventions and employed a broad spectrum of outcome measures [9–15]. For indirect comparison, we focus on the top 12 published studies ranked by quality in the recent systematic review by Van Thillo et al. [13], which comprised five randomized controlled trials (RCTs) [37–41] and seven non-randomized non-controlled trials (nRnCTs) [42–48]. Additionally, we chose two RCTs [49, 50] and one nRnCT [51] from the more recent systematic reviews [14, 15]. These 15 studies [37–51] recruited mostly PM/DM patients (except for two studies with IBM patients), 3–23 patients each, and used various inclusion criteria. The mean or median disease duration ranged from 2 months to 8.5 years, predominantly in the stable disease stage. The supervised intervention lasted from 2 to 12 weeks, 1–5 sessions weekly, 0.5–2 h per session, and focused on endurance or resistance training or a combination of both. The length of the follow-up period lasted from 0 to 44 or even 80 weeks [37–51].

Our study consisted of a 24-week supervised intervention (ADLRSp), twice weekly, 1 h per session, with a 30-min home-based exercise in the remaining 5 days of the week, and a 24-week follow-up. In our final analysis, 27 patients with a median disease duration of 6 years were included in the IG, and 23 patients with a median disease duration of 2.8 years were included in the CG. All our patients fulfilled the criteria either for PM/DM or for IMNM. Even though IBM was not an exclusion criterion, none of IBM patients enrolled into our study. A trend towards shorter disease duration in our CG, associated with significantly higher MMT-8ts, lower CK levels, and a higher current prednisone equivalent dose, might have been the underlying cause of the overall better baseline levels of most outcomes and their progressive deterioration observed mostly over weeks 0–24, since patients in earlier stages of IIM are more prone to rapid loss of muscle function [2, 3, 5]. However, this significant worsening was confirmed even after adjusting for baseline MMT-8ts, CK, and CPED. These findings further highlight the significance of non-pharmacological interventions early in the disease, to prevent the initial progressive deterioration of muscle function. Our study did not restrict the use of standard-of-care pharmacological therapy due to the very long duration of the supervised intervention and the character of the disease. However, no significant inter-group differences in the administration of DMARDs or glucocorticoids and their dosages were detected even after adjusting (data not shown). Nevertheless, the contribution of pharmacological therapy to the observed benefits of our ADLRSp in the IG cannot be estimated.

We employed several traditional primary (MMT-8, FI-2) and secondary (e.g., HAQ, SF-36, systemic measures of disease activity, Borg CR-10) outcomes, included some less frequently used ones (e.g., local measures of inflammation, fatigue, and depression), and investigated three new exploratory parameters derived from our preliminary data (i.e., stability, basal metabolism, and muscle fitness). In contrast to our approach, four [39, 40, 44, 51] out of 15 above-mentioned studies used the IMACS preliminary definitions of improvement with six core set measures [36]. Interestingly, three studies have demonstrated improvement according to IMACS response criteria in 6/9 [39], 7/11 [40], and 2/9 patients [44], even though none of these studies demonstrated any significant improvement in individually assessed MMT-8 or HAQ on a group level [39, 40, 44] with the exception of an improvement in MMT-8 in one study [40]. Since no significant change in CK levels was observed in any of these studies, we believe that the observed improvement according to IMACS response criteria in these studies [39, 40, 44] was based more on the subjective evaluation of global disease activity by patients and physicians using VAS and/or by MITAX assessing the extraskeletal disease activity. In our study, the data for MITAX and patient’s and physician’s global assessment of disease activity was only complete at baseline; thus, we could not perform the analysis according to the IMACS response criteria [36]. However, after applying a modified version of the 2016 ACR/EULAR response criteria [35] using the remaining three core set measures (MMT-8, HAQ, and CK), we demonstrated a moderate improvement over weeks 0–24 in the IG. In line with our findings of improvement in MMT-8, outcome measures assessing muscle strength improved to a different extent also in other mentioned studies, including MMT-8 in [37, 40], voluntary repetition maximum (VRM) in [40, 41], peak isometric torque (PIT) in [41, 48], leg press and/or leg extension and/or bench press in [42, 43, 51], or dynamometry of both distal and proximal muscles in [45]. Contrary to our study, no improvement in muscle strength was found according to MMT-8 in [39, 44, 49, 51], MMT on a scale from 0 to 20 in [48], or PIT in [50]. Similar to our findings, muscle endurance improved to a varying extent in four studies by Alexanderson et al., assessing FI-2 with improvement only in shoulder flexion in [44] or FI in [38, 46, 47]. Interestingly, clinically meaningful improvement (i.e., by >20%) in muscle endurance in two of these studies was detected in 7/10 [38] and 6/10 patients [47]. Regarding global function and disability, HAQ improved to a different extent in three studies [37, 42, 43], whereas no improvement was demonstrated in six studies [39, 40, 44, 45, 49, 51]. With respect to the quality of life, only two studies demonstrated an improvement in all eight domains of SF-36 [42] or in both PCS and MCS [43], whereas four studies [37, 40, 46, 47] detected an improvement only in some domains assessing the physical aspects, which is in line with a borderline improvement in PCS demonstrated in our study; two studies demonstrated no improvement in SF-36 [49, 50]. Fatigue assessed by VAS or Fatigue Severity Scale significantly decreased in one study [45], whereas it did not change in three other studies [35, 50, 52], which are in line with our findings. To our knowledge, depression has been examined by only one study [52], which used a Hospital Anxiety and Depression Scales and demonstrated no significant change. We believe that the significant mitigation of depression observed in our study is secondary to the functional improvement and sustained motivation thanks to the physiotherapist. In line with our findings, no significant change in perceived exertion or muscle pain during the intervention was found in four studies [39, 44, 45, 50]. Similarly, no increase in either one or several systemic markers of muscle damage or inflammation (mostly CK, LD, CRP, or ESR) was detected in any of the 15 above-mentioned studies [37–51]. To our knowledge, no previous study has evaluated the systemic levels or local mRNA levels of pro-inflammatory cytokines or chemokines. However, similar to our systemic findings, the study by Spector et al. [35] found no significant change in the number of leukocytes, epinephrine, cortisol, and lymphocyte subpopulations after their 12-week resistance training in IBM patients. Similarly, three studies by Alexanderson et al. [38, 44, 46] did not detect any increase in the inflammatory infiltrates in the muscle biopsy samples obtained after their 7-week, 12-week, and 24-week resistance training compared to baseline biopsy samples. Thus, our data on perceived exertion and measures of systemic and local inflammation further support the safety of non-pharmacological interventions in different subsets of IIM patients. The exploratory outcomes assessing stability (FVA), basal metabolism (BMR), and muscle fitness (ECM/BCM) can only be compared with studies on non-myositis populations since these parameters have never been evaluated in IIM patients to date. All three parameters were selected according to our preliminary analyses (unpublished data), which demonstrated the most significant and considerable differences between our IIM patients and age- and sex-matched healthy controls. BMR and ECM/BCM better reflect quantitative as well as qualitative changes in body compartments than traditional parameters such as body fat or lean body mass [25, 27, 53–55]. Similar to our findings in IIM patients, stability training improved the standing stability also in neuropathic patients with type 2 diabetes [56] and in hemiplegic stroke patients [57]. In accordance with our findings, a 10-week concurrent resistance and endurance training, as well as merely a resistance training, increased basal metabolism (BMR) in healthy young men [58]. Similarly, a stabilization of muscle fitness (ECM/BCM) from a 12-month guided exercise program was observed in patients with chronic obstructive pulmonary disease at months 6 and 12 of the study by Jungblut et al., compared to progressive deterioration in those who did not exercise [59]. However, our findings on stability, basal metabolism, and muscle fitness need to be interpreted with caution due to the borderline significant changes observed only after the first 12 weeks of our ADLRSp, probably due to an initiation effect, and thus deserve evaluation and confirmation in further studies. To conclude our observations, our recently published in vitro study on skeletal muscle cells [60], derived from the muscle biopsy samples of our IIM volunteers from the IG at baseline and after the 24-week ADLRSp, might shed some light on the possible mechanism underlying the observed functional improvements in our study. Based on these in vitro data, our ADLRSp improved or normalized intrinsic metabolic defects represented by altered dynamics of lipid metabolism and impaired adaptive response to saturated fatty acid load [60].

Our study has a few limitations. Firstly, it was a non-randomized controlled study. We could not perform a randomized trial due to the length of supervised intervention (24 weeks), extended follow-up (24 weeks), and a large recruitment target of patients (*n* = 25–30 per group) for a rare disease. Our IG cohort was thus limited to those living within the vicinity of Prague, or possibly those with better overall function and milder fatigue and better accessibility to our institute. The potential selection and allocation bias was addressed by consecutive non-selective recruitment of all treated IIM patients fulfilling the inclusion criteria at our center from 2014 to 2016, by using assessors blinded to the group allocation, and by adjusting for significantly different baseline variables between the IG and CG. Secondly, the sample size precluded subgroup analysis to identify individuals with the largest benefit. Further studies are required to confirm the efficacy of our ADLRSp specifically in early disease stages or an inception cohort, or in patients with a recent flare. Thirdly, the concurrent standard-of-care pharmacological therapy was indispensable due to the exceptionally long duration of the study and the nature of the disease. More importantly, we aimed to provide evidence for our ADLRSp under a clinical setting for practical routine use. Lastly, given the combination of several different intervention methods, the major contribution of any particular technique to the overall improvement can hardly be assessed. On the other hand, judging by the duration of individual components of the 24-week ADLRSp (training of ADL 83%, resistance 13%, and stability 4%), the substantial proportion of achieved effects may be attributed to the training of ADL.

## Conclusions

Our 24-week supervised, tailored program for IIM patients focused on activities of daily living, resistance, and stability (ADLRSp) training not only pre-empted the progressive deterioration of the muscle function observed in the control group, but also resulted in a significant improvement in muscle strength and endurance, global disability, depression, stability, and basal metabolism, as well as stabilization of muscle fitness. The achieved improvement in muscle strength and endurance, global disability, and stability was clinically meaningful in a substantial proportion of IIM patients. Although the improvement in most outcomes in the intervention group was still present at the end of the follow-up period, the maximum effects were not sustained, which underlines the need for regular professional supervision and motivation of patients. This ADLRSp was safe and did not aggravate the disease activity. Our findings provide substantial evidence of considerably improved functional outcome upon training of activities of daily living, in combination with resistance and stability training, derived from a robust number of IIM patients from routine clinical practice, and further support the crucial role of non-pharmacological interventions in multidisciplinary care for patients with IIM. These data emphasize the need for early initiation of continuous and regularly supervised exercise, in order to prevent the progressive loss of function and to mitigate the already established disability, thereby improving the quality of life of patients with IIM.

## Supplementary Information

**Additional file 1.** Methods.

**Additional file 2: Table S1.** Primary and secondary outcomes assessing muscle strength, endurance, global function/disability, depression, stability, metabolism and muscle fitness. **Table S2.** Primary and secondary outcomes assessing muscle strength in individual muscle groups, fatigue, and quality of life. **Table S3.** Perceived exertion and disease activity assessed by systemic levels of markers for inflammation and muscle damage. **Table S4.** Relative mRNA expression of pro-inflammatory cytokines and chemokines in muscle biopsy prior and after the intervention (*n*=7).

## Data Availability

Individual anonymized participant data will not be shared. Pooled study data, protocol, or statistical analysis plan can be shared upon request at tomcik@revma.cz.

## Abbreviations

IIM: Idiopathic inflammatory myopathies
ADL: Activities of daily living
IG: Intervention group
CG: Control group
MMT-8: Manual Muscle Testing of eight muscle groups
FI-2: Functional Index-2
HAQ: Health Assessment Questionnaire
SF-36: Medical Outcomes Study 36-item Short Form Health Survey
BDI-II: Beck’s Depression Inventory-II
FIS: Fatigue Impact Scale
DM: Dermatomyositis
PM: Polymyositis
IBM: Inclusion body myositis
CADM: Clinically amyopathic dermatomyositis
ASS: Antisynthetase syndrome
IMNM: Immune-mediated necrotizing myopathy
ADLRSp: Program focused on activities of daily living, resistance, and stability training
ENMC: European Neuromuscular Centre
MMT-8ts: Total score of MMT-8
PCS: Physical component score
MCS: Mental component score
FVA: Force vector area
BMR: Basal metabolic rate
ECM/BCM: Extracellular mass to body cell mass ratio
Borg CR-10: Borg Category-Ratio-10 perceived exertion scale
ESR: Erythrocyte sedimentation rate
CRP: C-reactive protein
CK: Creatine kinase
LD: Lactate dehydrogenase
IL: Interleukin
TNF: Tumor necrosis factor
MCP-1: Monocyte chemoattractant protein-1
CCL-2: Chemokine (C-C motif) ligand 2
mRNA: Messenger ribonucleic acid
ACR: American College of Rheumatology
EULAR: European League Against Rheumatism
TIS: Total improvement score
CPED: Current prednisone equivalent dose
DMARDs: Disease-modifying antirheumatic drugs
SEM: Standard error of the mean
IQR: Inter-quartile range
ANOVA: Analysis of variance
LSD: Fisher’s least significant difference
PRO: Patient-reported outcomes
IMACS: International Myositis Assessment and Clinical Studies Group
MITAX: Myositis Intent-to-Treat Activity Index
VRM: Voluntary repetition maximum
PIT: Peak isometric torque
LBM: Lean body mass
BF: Body fat
BCM: Body cell mass
ECM: Extracellular mass
HC: Healthy controls
RNA: Ribonucleic acid
ACTB: Beta-actin
RPL13: A gene encoding the 60S ribosomal protein L13
RS: Resistance and stability training
DNS: Dynamic neuromuscular stabilization
PNF: Proprioceptive neuromuscular facilitation
VAS: Visual analog scale
RPE: Rating of perceived exertion
VO2max: Maximum rate of oxygen consumption
