# Supplementary material for: The effect of a 24-week training focused on activities of daily living, muscle strengthening, and stability in idiopathic inflammatory myopathies: a monocentric controlled study with follow-up
Source: Arthritis Res Ther. 2021 Jun 21;23:173. doi: 10.1186/s13075-021-02544-5 (PMC8218432; doi:10.1186/s13075-021-02544-5)
Supplement: Supplementary file 1 — Additional file 1. Methods. [file 13075_2021_2544_MOESM1_ESM.docx]

**Supplementary Material**

**METHODS**

**Primary outcome measures**

All objective outcome measures were evaluated in both the intervention (IG) and control group (CG) at the same time of day by a trained assessor, physiotherapist blinded to group allocation and the treatment of IIM patients.

Manual Muscle Testing-8 (MMT-8) was used for the evaluation of isometric strength of eight muscle groups (on the dominant side only) on a scale ranging from 0 (no muscle contraction) to 10 (holding the test position against strong resistance). The MMT-8 total score (MMT-8ts) ranges from 0 (minimum) to 80 (maximum) ([1](#_ENREF_1)). In addition, MMT was also assessed in m. triceps brachii and m. iliopsoas.

Functional index-2 (FI-2) was used to evaluate muscle endurance in seven muscle groups with a score ranging from 0% (minimum) to 100% (maximum) ([1](#_ENREF_1)). The mean of these seven muscle group scores was used for the analysis, ranging from 0-100%. The Borg Category-Ratio (CR)-10 perceived exertion scale with a score ranging from 0 (no exertion) to 10 (almost maximum exertion) ([2](#_ENREF_2), [3](#_ENREF_3)) was used concurrently with FI-2, in order to assess safety.

**Secondary outcome measures**

*Patient reported outcomes*

Functional status was assessed by the Health Assessment Questionnaire (HAQ), evaluating patients’ ability to perform various activities of daily living in eight domains with a final score graded from 0 (no difficulty) to 3 (unable to perform the task) ([4](#_ENREF_4)). HAQ has been used for IIM patients ([1](#_ENREF_1)) and was previously translated and validated in Czech ([5](#_ENREF_5)).

The Medical Outcomes Study 36-item Short Form Health Survey (SF-36) was used to assess the quality of life ([6](#_ENREF_6)). SF-36 contains eight domains graded from 0 (worst score) to 100 (best score) and can be summarized in two aggregate scores: the physical component score (PCS) and the mental component score (MCS) ([6](#_ENREF_6)), which, for this study, were calculated from a normative sample of the general adult Czech population ([7](#_ENREF_7)) and factor score coefficients ([8](#_ENREF_8)). SF-36 has been used for IIM ([9](#_ENREF_9)) and was previously validated for use in Czech ([7](#_ENREF_7)).

Fatigue was assessed by the Fatigue Impact Scale (FIS) assessing the impact of fatigue on patients’ cognitive, physical, and psychosocial function. The total score ranges from 0 (no fatigue) to 160 (maximum impact of fatigue) ([10](#_ENREF_10)). FIS has been translated and validated for use in Czech ([11](#_ENREF_11)).

The intensity and frequency of depressive symptoms were evaluated by the second edition of Beck’s Depression Inventory (BDI-II) ([12](#_ENREF_12)). Patients respond to 21 items using a four-point scale graded from 0 (no depression) to 3 (most severe depression) with a total score of 0-63. BDI-II has demonstrated excellent psychometric properties in various conditions ([13](#_ENREF_13)), and has been translated and validated for use in Czech ([14](#_ENREF_14)).

*Evaluation of treatment response according to the 2016 ACR/EULAR response criteria*

The final myositis response criteria for minimal, moderate, and major improvement in adult DM/PM clinical trials and studies were proposed by ACR/EULAR in 2016 ([15](#_ENREF_15)). Briefly, the criteria include the absolute percentage changes in six core set measures: physician's and patient's global activity, MMT, HAQ, most abnormal enzyme levels, and extramuscular disease activity. The Total Improvement Score (TIS) is the sum of the scores for these six core set measures, ranging from 0 (no improvement) to 100 (maximum improvement) with three levels of improvement: minimal (≥20 points), moderate (≥40 points), and major (≥60 points). Since our data on physician's and patient's global activity and extramuscular disease activity were incomplete at weeks 12, 24, and 48, we calculated the individual scores only for three core set measures (MMT-8, HAQ, and CK), and assessed the percentage distribution of patients in these three levels of improvement, with one additional level of “least improvement (0-19 points) and worsening”. Since the TIS calculated from MMT-8, HAQ, and CK can only reach a maximum score of 50, we adjusted the threshold values for levels of improvement accordingly, i.e., minimal improvement (10-19 points), moderate improvement (20-29 points) and major improvement (≥30 points), with the additional level of “least improvement (0-9 points) and worsening”, and assessed the percentage distribution of patients respectively.

*Assessment of stability*

Stability was assessed on the ground force reaction platform Balance‐X‐Sensor (Balance-X-Sensor Pro 7860.20.001, Soehnle Professional GmbH and Co. KG, Murrhardt, Germany) by performing a three standing‐exercise test with the eyes open and then closed, as described elsewhere ([16](#_ENREF_16), [17](#_ENREF_17)). Since our previous study demonstrated the most significant difference between IIM patients and healthy controls (HC) (p=0.009) in force vector area (FVA, cm^2^) assessed by Tandem stand (i.e., holding a position with the heel to toe without taking a supporting step) with eyes closed (i.e., the hardest exercise possible), we hypothesized that this parameter would be most sensitive to change. FVA represents the balance area of the center of gravity of the body, and a small area indicates a high degree of stability ([17](#_ENREF_17), [18](#_ENREF_18)).

*Assessment of basal metabolism and muscle fitness*

Our recent study on body composition (manuscript in preparation) demonstrated the most considerable and significant differences in two complex measures reflecting the changes in different body compartments: compared to HC, IIM patients had a significantly decreased basal metabolic rate (BMR) (p<0.0001) and an increased extracellular mass to body cell mass ratio (ECM/BCM) (p<0.0001), which represents worse muscle fitness or deteriorated fitness for physical exercise ([19](#_ENREF_19)). BMR is the daily rate of energy metabolism to preserve the integrity of vital functions, including breathing, blood circulation, maintaining body temperature, cell growth, brain and nerve function, and contraction of muscles, which accounts for 18% of the total energy expenditure ([20](#_ENREF_20)). BCM consists of metabolically active organs and particularly muscles ([21](#_ENREF_21), [22](#_ENREF_22)), and is responsible for changes in BMR ([23](#_ENREF_23)). ECM includes connective tissues such as collagen, elastin, skin, tendons, and bones, as well as interstitial fluid. In healthy individuals of middle age, the BCM is always higher than the ECM; thus, the ECM/BCM ratio is <1 ([21](#_ENREF_21), [22](#_ENREF_22)). The ECM/BCM ratio reflects quantitative and qualitative changes in muscle mass and can be used for assessing muscle fitness or fitness for exercise ([24](#_ENREF_24)). Therefore, we hypothesized that BMR and ECM/BCM, out of all body composition parameters assessed by bioelectric impedance analysis (BIA), might be most sensitive to change. The body composition variables were measured using a multi-frequency bioelectrical impedance analyzer (BIA-2000M, Data Input GmbH, Pöcking, Germany) according to the manufacturer’s instructions and a standardized protocol ([25](#_ENREF_25)), as described elsewhere ([26](#_ENREF_26)).

*Assessment of proinflammatory cytokines and chemokines in muscle tissue*

A Bergström needle biopsy of m. vastus lateralis on seven volunteers from the IG at week 0 and 24, after an overnight fasting under aseptic conditions with local anaesthesia, was performed as described elsewhere ([27](#_ENREF_27)). The post-intervention biopsy was taken within a month (mean ± standard error of the mean: 20.0 ± 9.1 days) from the last supervised ADLRSp session. Muscle samples were immediately frozen in liquid nitrogen and were stored at -80°C until RNA isolation. Isolation of total RNA, testing for concentration and purity, reverse transcription, and the analysis of the gene expression of IL-1β, IL-6, IL-8, TNF, MCP-1, ACTB, and RPL13 were performed as described in our previous study ([28](#_ENREF_28)). The data on mRNA expression are presented as relative x-fold change with pre-intervention values set at 1.

**Intervention program**

In the IG, an individually personalized 24-week program (ADLRSp) focused on training of activities of daily living (ADL, the first day) and resistance and stability training (RS, the second day), one hour per session, was conducted by the same physiotherapist (MS) experienced in treating IIM patients. The educational material on home-based exercise contained the same exercises as the supervised ADL session, condensed to 30 min. The IG was instructed to perform the home-based exercise in the remaining five days of the week over weeks 0-24, and daily over weeks 24-48. Whereas the CG was instructed to perform the home-based exercise daily over weeks 0-48, which represents a non-pharmacological standard of care.

Each supervised session started with a warm-up activity (stair climbing, treadmill, stationary bicycle, step-up) for 10 min, and concluded with stretching and breathing relaxation for 10 min.

The supervised ADL training session (40 min) included:

a) elements from Dynamic Neuromuscular Stabilization (DNS) ([18](#_ENREF_18)) based on the principles of developmental kinesiology;

b) “stork walk” or “marching” (standing and sitting position) and training of putting on socks;

c) training in handling of loads (standing and sitting position using an exercise ball or different loads) using Proprioceptive Neuromuscular Facilitation (PNF) ([29](#_ENREF_29));

d) training of hanging clothes, combing, putting on brassieres (using an exercise ball or different loads) in standing or supine position;

e) isometric strengthening of hip joint adductors (using an exercise ball or a gym ball).

The supervised RS training session (40 min) focused on the resistance training of the weakened muscle groups for 30 min and stability training using the ProprioSphere device (Kinetec, Aldershot, UK) for 10 min. The resistance training was performed according to the following rules:

a) 50-69% of maximum voluntary contraction;

b) 10-15 repetitions (for adults), 8-12 repetitions (for elderly);

c) three sets;

d) 1-2 min break between the sets.

**Safety and adherence monitoring**

The safety of the supervised ADLRSp was monitored on several levels, and the intensity was adapted accordingly:

a) Visual Analog Scales (VAS) for pain, dyspnea, and fatigue,

b) the ability to speak during exercise using the talk test ([30](#_ENREF_30)),

c) maintaining a moderate amount of workload (i.e., 55-69% of the maximum heart rate [220–age]) ([31](#_ENREF_31)),

d) Borg Rating of Perceived Exertion (RPE) scale ([2](#_ENREF_2)), graded from 6 (extremely low exertion) to 20 (extremely high exertion), maintaining values between 12-13, which correspond to moderate exercise intensity and 40-59% (for adults) and 32-47% (for elderly) of VO2max (maximum rate of oxygen consumption) ([31](#_ENREF_31)).

The adherence was evaluated by recording attendance and progress. The daily home exercise was monitored by a diary with recorded performance of exercises, and a semi-quantitative assessment of effort, pain, and dyspnea before and after exercise using VAS. In the IG, the diaries were evaluated by a physiotherapist (MS) weekly over weeks 0-24 and at week 48. In the CG, the diaries were evaluated at weeks 0, 12, 24, and 48.

**Clinical assessments**

At baseline, all patients underwent clinical examination by a physician experienced in treating IIM patients (JV, HM), blinded to the non-pharmacological intervention, and were assessed according to international guidelines ([32](#_ENREF_32), [33](#_ENREF_33)), as described in our previous study ([28](#_ENREF_28)). All individual treatments by rheumatologists, physical therapists, and nurses during the duration of the study were recorded into the medical files.

**Laboratory assessment**

At baseline, 12, 24, and 48 weeks, all patients provided peripheral blood for routine biochemistry, full blood count, and biobanking. Serum levels of C-reactive protein (CRP), creatine phosphokinase (CK), lactate dehydrogenase (LD), and myoglobin, as well as the assessment of autoantibodies were performed as described in our previous study ([34](#_ENREF_34)). The plasma concentrations of selected cytokines/chemokines were measured by a commercially available Bio-Plex ProTM human Cytokine 27-plex Assay (BIO-RAD, California, USA) according to the manufacturer’s instruction, as described in our previous study ([28](#_ENREF_28)).

**REFERENCES**

1. Rider LG, Werth VP, Huber AM, Alexanderson H, Rao AP, Ruperto N, et al. Measures of adult and juvenile dermatomyositis, polymyositis, and inclusion body myositis: Physician and Patient/Parent Global Activity, Manual Muscle Testing (MMT), Health Assessment Questionnaire (HAQ)/Childhood Health Assessment Questionnaire (C-HAQ), Childhood Myositis Assessment Scale (CMAS), Myositis Disease Activity Assessment Tool (MDAAT), Disease Activity Score (DAS), Short Form 36 (SF-36), Child Health Questionnaire (CHQ), physician global damage, Myositis Damage Index (MDI), Quantitative Muscle Testing (QMT), Myositis Functional Index-2 (FI-2), Myositis Activities Profile (MAP), Inclusion Body Myositis Functional Rating Scale (IBMFRS), Cutaneous Dermatomyositis Disease Area and Severity Index (CDASI), Cutaneous Assessment Tool (CAT), Dermatomyositis Skin Severity Index (DSSI), Skindex, and Dermatology Life Quality Index (DLQI). Arthritis Care Res (Hoboken). 2011;63 Suppl 11:S118-57.

2. Borg GA. Psychophysical bases of perceived exertion. Med Sci Sports Exerc. 1982;14(5):377-81.

3. Noble BJ, Borg GA, Jacobs I, Ceci R, Kaiser P. A category-ratio perceived exertion scale: relationship to blood and muscle lactates and heart rate. Med Sci Sports Exerc. 1983;15(6):523-8.

4. Bukhari M, Kent A. How rheumatologists assess disability in the current era needs an overhaul: focus on the Health Assessment Questionnaire. Rheumatology (Oxford). 2020;59(2):267-8.

5. Šléglová O, Dušek L, Olejárová M, Tegzová D, Vencovský J, Pavelka K. Assessment of functional ability in patients with rheumatoid arthritis; validation of the Czech version of the Stanford Health Assessment Questionnaire (HAQ). Ces Revmatol. 2010;18(2):73-83.

6. Ware JE, Jr., Sherbourne CD. The MOS 36-item short-form health survey (SF-36). I. Conceptual framework and item selection. Med Care. 1992;30(6):473-83.

7. Sobotík Z. Zkušenosti s použitím předběžné české verze amerického dotazníku o zdraví (SF 36). Zdravotnictví v České republice. 1998;1(1-2):50-4.

8. Ware JJ, Kosinski M, Keller S. SF-36 physical and mental health summary scales: a user's manual. Health Assessment Lab.1994.

9. Miller FW, Rider LG, Chung YL, Cooper R, Danko K, Farewell V, et al. Proposed preliminary core set measures for disease outcome assessment in adult and juvenile idiopathic inflammatory myopathies. Rheumatology (Oxford). 2001;40(11):1262-73.

10. Fisk JD, Ritvo PG, Ross L, Haase DA, Marrie TJ, Schlech WF. Measuring the functional impact of fatigue: initial validation of the fatigue impact scale. Clin Infect Dis. 1994;18 Suppl 1:S79-83.

11. Heřmánková B, Šmucrová H, Mikulášová M, Oreská S, Špiritović M, Štorkánová H, et al. Validation of Czech versions of questionnaires assessing fatigue and physical activity in patients with rheumatic diseases: Fatigue Impact Scale (FIS), Multidimensional Assessment of Fatigue Scale (MAF), Human Activity Profile (HAP). Ces revmatol. 2020;28(3):132-51.

12. Beck A, Steer R, Brown G. Manual for the beck depression inventory-II. San Antonio, TX: Psychological Corporation; 1996;1:82.

13. Wang YP, Gorenstein C. Psychometric properties of the Beck Depression Inventory-II: a comprehensive review. Braz J Psychiatry. 2013;35(4):416-31.

14. Ptáček R, Raboch J, Vňuková M, Hlinka J, Anders M. Beckova škála deprese BDI-II - standardizace a využití v praxi. Ceska a Slovenska Psychiatrie. 2016;112:270-4.

15. Aggarwal R, Rider LG, Ruperto N, Bayat N, Erman B, Feldman BM, et al. 2016 American College of Rheumatology/European League Against Rheumatism criteria for minimal, moderate, and major clinical response in adult dermatomyositis and polymyositis: An International Myositis Assessment and Clinical Studies Group/Paediatric Rheumatology International Trials Organisation Collaborative Initiative. Ann Rheum Dis. 2017;76(5):792-801.

16. Schneider P, Hänscheid H, Schwab M, Jakob F. Assessment of neuromuscular function with a new ground reaction force platform using power spectrum analysis technique. In: Dössel O, Schlegel W, editors. World Congress on Medical Physics and Biomedical Engineering, September 7 - 12, 2009, Munich, Germany. Heidelberg: Springer Berlin; 2009; Vol. 25/12. p. 1-4.

17. Refardt J, Kling B, Krausert K, Fassnacht M, von Felten S, Christ-Crain M, et al. Impact of chronic hyponatremia on neurocognitive and neuromuscular function. Eur J Clin Invest. 2018;48(11):e13022.

18. Frank C, Kobesova A, Kolar P. Dynamic neuromuscular stabilization & sports rehabilitation. Int J Sports Phys Ther. 2013;8(1):62-73.

19. Bunc V, Dlouha R, Moravcova J, Novak I, Hoskova Z, Cermakova M. Estimation of body composition by multifrequency bioimpedance measurement in children. Ann N Y Acad Sci. 2000;904:203-4.

20. Henry CJ. Basal metabolic rate studies in humans: measurement and development of new equations. Public Health Nutr. 2005;8(7A):1133-52.

21. Staufer K, Halilbasic E, Hillebrand P, Harm S, Schwarz S, Jaksch P, et al. Impact of nutritional status on pulmonary function after lung transplantation for cystic fibrosis. United European Gastroenterol J. 2018;6(7):1049-55.

22. Talluri T, Lietdke RJ, Evangelisti A, Talluri J, Maggia G. Fat-free mass qualitative assessment with bioelectric impedance analysis (BIA). Ann N Y Acad Sci. 1999;873:94-8.

23. Abdel-Mageed S, Mohamed E. Total Body Capacitance for Estimating Human Basal Metabolic Rate in an Egyptian Population. Int J Biomed Sci. 2016;12(1):42-7.

24. Roche A, Heymsfield S, Lohman T. Human body composition. Champaign IL: Human Kinetics Publishers; 1996.

25. Kyle UG, Bosaeus I, De Lorenzo AD, Deurenberg P, Elia M, Manuel Gomez J, et al. Bioelectrical impedance analysis-part II: utilization in clinical practice. Clin Nutr. 2004;23(6):1430-53.

26. Bunc V, Hráský P, Skalská M. Body Composition and Aerobic Fitness like a Result of 6 Months Walking Program in Senior Women. JMED Research. 2014:950910.

27. Kurdiova T, Balaz M, Vician M, Maderova D, Vlcek M, Valkovic L, et al. Effects of obesity, diabetes and exercise on Fndc5 gene expression and irisin release in human skeletal muscle and adipose tissue: in vivo and in vitro studies. J Physiol. 2014;592(5):1091-107.

28. Kropackova T, Vernerova L, Storkanova H, Horvathova V, Vokurkova M, Klein M, et al. Clusterin is upregulated in serum and muscle tissue in idiopathic inflammatory myopathies and associates with clinical disease activity and cytokine profile. Clin Exp Rheumatol. 2020.

29. Kabat H, Mc LM, Holt C. The practical application of proprioceptive neuromuscular facilitation. Physiotherapy. 1959;45(4):87-92.

30. Foster C, Porcari JP, Anderson J, Paulson M, Smaczny D, Webber H, et al. The talk test as a marker of exercise training intensity. J Cardiopulm Rehabil Prev. 2008;28(1):24-30; quiz 1-2.

31. Fletcher GF, Balady GJ, Amsterdam EA, Chaitman B, Eckel R, Fleg J, et al. Exercise standards for testing and training: a statement for healthcare professionals from the American Heart Association. Circulation. 2001;104(14):1694-740.

32. Miller FW. New approaches to the assessment and treatment of the idiopathic inflammatory myopathies. Ann Rheum Dis. 2012;71 Suppl 2:i82-5.

33. Isenberg DA, Allen E, Farewell V, Ehrenstein MR, Hanna MG, Lundberg IE, et al. International consensus outcome measures for patients with idiopathic inflammatory myopathies. Development and initial validation of myositis activity and damage indices in patients with adult onset disease. Rheumatology (Oxford). 2004;43(1):49-54.

34. Mann H, Krystufkova O, Zamecnik J, Hacek J, Hulejova H, Filkova M, et al. Interleukin-35 in idiopathic inflammatory myopathies. Cytokine. 2021;137:155350.
