# Supplementary material for: The effect of a 24-week training focused on activities of daily living, muscle strengthening, and stability in idiopathic inflammatory myopathies: a monocentric controlled study with follow-up
Source: Arthritis Res Ther. 2021 Jun 21;23:173. doi: 10.1186/s13075-021-02544-5 (PMC8218432; doi:10.1186/s13075-021-02544-5)
Supplement: Supplementary file 2 — Additional file 2: Table S1. Primary and secondary outcomes assessing muscle strength, endurance, global function/disability, depression, stability, metabolism and muscle fitness. Table S2. Primary and secondary outcomes assessing muscle strength in individual muscle groups, fatigue, and quality of life. Table S3. Perceived exertion and disease activity assessed by systemic levels of markers for inflammation and muscle damage. Table S4. Relative mRNA expression of pro-inflammatory cytokines and chemokines in muscle biopsy prior and after the intervention (n=7). [file 13075_2021_2544_MOESM2_ESM.docx]

|  |  | **Control group** | | | |  | **Intervention group** | | | |  |  |
| --- | --- | --- | --- | --- | --- | --- | --- | --- | --- | --- | --- | --- |
|  |  |  |  | **Intra-group analysis** | |  |  |  | **Intra-group analysis** | | **Inter-group analysis** | |
|  |  |  |  | **unadjusted** | **adjusted** |  |  |  | **unadjusted** | **adjusted** | **unadjusted** | **adjusted** |
| **Parameter**  (score range worst-best) | **time** | **median (IQR)** |  | **p-value** | **p-value** |  | **median (IQR)** |  | **p-value** | **p-value** | **p-value** | **p-value** |
| **MMT-8 total score**  (0-80) | 1=w0 | 64.0 (55.0 - 71.0) | p^12^ | **<0.0001** | **-** |  | 56.0 (48.0 - 65.0) | p^12^ | **<0.0001** | **-** | **<0.0001** | **-** |
|  | 2=w12 | 58.0 (52.0 - 65.0) | p^23^ | **0.0079** | **-** |  | 63.0 (57.0 - 69.0) | p^23^ | **<0.0001** | **-** |  |  |
|  | 3=w24 | 55.0 (45.0 - 60.0) | p^13^ | **<0.0001** | **-** |  | 72.0 (64.0 - 76.0) | p^13^ | **<0.0001** | **-** |  |  |
|  | 4=w48 | 58.0 (48.0 - 64.0) | p^34^ | **0.0383** | **-** |  | 67.0 (53.0 - 74.0) | p^34^ | **0.0002** | **-** |  |  |
| **FI-2**  (0-100) | 1=w0 | 34.3 (12.8 - 59.1) | p^12^ | **0.0155** | **0.0424** |  | 25.9 (10.4 - 49.2) | p^12^ | **<0.0001** | **<0.0001** | **<0.0001** | **<0.0001** |
|  | 2=w12 | 23.6 (9.5 - 39.2) | p^23^ | 0.0696 | 0.2532 |  | 47.0 (29.6 - 67.1) | p^23^ | **<0.0001** | **<0.0001** |  |  |
|  | 3=w24 | 27.3 (13.0 - 34.8) | p^13^ | **0.0019** | **0.0064** |  | 76.1 (56.8 - 93.7) | p^13^ | **<0.0001** | **<0.0001** |  |  |
|  | 4=w48 | 26.7 (11.7 - 41.1) | p^34^ | 0.8665 | 0.3542 |  | 66.7 (29.5 - 84.1) | p^34^ | **<0.0001** | **0.0010** |  |  |
| **HAQ**  (3-0) | 1=w0 | 1.25 (0.63 - 1.88) | p^12^ | **0.0373** | 0.1656 |  | 0.50 (0.25 - 1.63) | p^12^ | **0.0096** | **0.0014** | **0.0002** | **<0.0001** |
|  | 2=w12 | 1.25 (0.75 - 2.25) | p^23^ | 1.0000 | 0.0958 |  | 0.38 (0.00 - 1.13) | p^23^ | **0.0141** | **0.0348** |  |  |
|  | 3=w24 | 1.25 (0.75 - 2.38) | p^13^ | 0.1663 | **0.0341** |  | 0.25 (0.00 - 1.00) | p^13^ | **0.0010** | **0.0001** |  |  |
|  | 4=w48 | 1.50 (0.63 - 2.25) | p^34^ | 0.2047 | 0.7672 |  | 1.00 (0.13 - 1.25) | p^34^ | **0.0008** | **0.0111** |  |  |
| **BDI-II**  (63-0) | 1=w0 | 12 (7 - 18) | p^12^ | 0.3809 | 0.8406 |  | 9 (6 - 15) | p^12^ | 0.2693 | 0.7258 | **0.0025** | **0.0366** |
|  | 2=w12 | 13 (8 - 24) | p^23^ | 0.3177 | 0.2906 |  | 9 (5 - 16) | p^23^ | **0.0159** | 0.0571 |  |  |
|  | 3=w24 | 16 (13 - 20) | p^13^ | 0.0529 | 0.3547 |  | 8 (3 - 11) | p^13^ | **0.0029** | **0.0451** |  |  |
|  | 4=w48 | 14 (8 - 24) | p^34^ | 0.8615 | 0.9489 |  | 9 (3 - 14) | p^34^ | **0.0415** | 0.6657 |  |  |
| **Force vector area (cm^2^)**  (57-0) | 1=w0 | 7.0 (5.2 - 9.4) | p^12^ | **0.0488** | 0.1491 |  | 7.0 (4.0 - 12.5) | p^12^ | **0.0303** | 0.0623 | **0.0081** | **0.0373** |
|  | 2=w12 | 8.1 (5.5 - 21.0) | p^23^ | 0.1767 | 0.3743 |  | 5.1 (2.9 - 6.9) | p^23^ | 0.1767 | 0.1760 |  |  |
|  | 3=w24 | 7.4 (5.8 - 13.8) | p^13^ | 0.1584 | 0.2160 |  | 4.2 (2.4 - 9.9) | p^13^ | 0.6398 | 0.8013 |  |  |
|  | 4=w48 | 8.6 (6.3 - 16.1) | p^34^ | 0.3214 | 0.5235 |  | 4.5 (3.0 - 8.5) | p^34^ | 0.4345 | 0.3486 |  |  |
| **BMR (kcal/day)**  (900-1900) | 1=w0 | 1230 (1173 - 1375) | p^12^ | **0.0216** | **0.0052** |  | 1220 (1120 - 1338) | p^12^ | **0.0262** | 0.1011 | **0.0476** | 0.0539 |
|  | 2=w12 | 1215 (1113 – 1335) | p^23^ | 0.1057 | 0.5396 |  | 1270 (1165 - 1345) | p^23^ | 0.8297 | 0.5036 |  |  |
|  | 3=w24 | 1215 (1140 - 1358) | p^13^ | 0.3392 | **0.0292** |  | 1275 (1160 - 1360) | p^13^ | 0.1283 | 0.0673 |  |  |
|  | 4=w48 | 1225 (1153 - 1350) | p^34^ | 0.9701 | 0.7284 |  | 1275 (1148 - 1335) | p^34^ | 0.2094 | 0.8697 |  |  |
| **ECM/BCM**  (3.1-0.8) | 1=w0 | 1.34 (1.19 - 1.56) | p^12^ | **0.0077** | **0.0009** |  | 1.35 (1.21 - 1.63) | p^12^ | 0.6473 | 0.8667 | 0.0500 | 0.1032 |
|  | 2=w12 | 1.43 (1.29 - 1.91) | p^23^ | 0.3928 | 0.8726 |  | 1.31 (1.13 - 1.63) | p^23^ | 0.6024 | 0.2335 |  |  |
|  | 3=w24 | 1.37 (1.25 – 1.96) | p^13^ | 0.0506 | **0.0015** |  | 1.34 (1.12 - 1.54) | p^13^ | 0.4117 | 0.3154 |  |  |
|  | 4=w48 | 1.41 (1.25 - 1.61) | p^34^ | 0.0988 | 0.0860 |  | 1.33 (1.12 - 1.61) | p^34^ | 0.5992 | 0.4244 |  |  |
| **Acronyms:** IQR, inter-quartile range; MMT-8, Manual Muscle Testing of eight muscles; FI-2, Functional Index 2; HAQ, Health Assessment Questionnaire; BDI-II, Beck's Depression Inventory II; BMR, basal metabolic rate; ECM/BCM, extracellular mass to body cell mass ratio; w0, week 0 (baseline); w12, week 12 (after 12 weeks of intervention); w24, week 24 (after 24 weeks of intervention); w48, week 48 (24 weeks after the end of intervention); p^12^ – difference between time 1 and 2; p^23^ – difference between time 2 and 3; p^13^ – difference between time 1 and 3; p^34^ – difference between time 3 and 4; Unadjusted data are presented as median (IQR). Statistically significant differences (p<0.05) are marked in bold. Intra-group comparisons were performed by one way ANOVA. Inter-group comparisons were performed by two way ANOVA. P-values are provided for both unadjusted data and for data adjusted for MMT-8, creatine kinase and the current prednisone equivalent dose. | | | | | | | | | | | | |

**Table S1. Primary and secondary outcomes assessing muscle strength, endurance, global function/disability, depression, stability, metabolism and muscle fitness**

**Table S2. Primary and secondary outcomes assessing muscle strength in individual muscle groups, fatigue, and quality of life**

|  |  | **Control group** | | | |  | **Intervention group** | | | |  |  | |
| --- | --- | --- | --- | --- | --- | --- | --- | --- | --- | --- | --- | --- | --- |
|  |  |  |  | **Intra-group analysis** | |  |  |  | **Intra-group analysis** | | **Inter-group analysis** | | |
|  |  |  |  | **unadjusted** | **adjusted** |  |  |  | **unadjusted** | **adjusted** | **unadjusted** | **adjusted** | |
| **Parameter**  (score range worst-best) | **time** | **median (IQR)** |  | **p-value** | **p-value** |  | **median (IQR)** |  | **p-value** | **p-value** | **p-value** | **p-value** | |
| **MMT-8**  **deep neck flexors**  (0-10) | 1=w0 | 7.0 (6.0 - 8.0) | p^12^ | **0.0013** | **0.0462** |  | 5.0 (3.0 - 7.0) | p^12^ | **<0.0001** | **<0.0001** | **<0.0001** | **<0.0001** | |
|  | 2=w12 | 6.0 (5.0 - 7.0) | p^23^ | 0.1481 | 0.9804 |  | 6.0 (5.0 - 8.0) | p^23^ | **<0.0001** | **0.0012** |  |  |  |
|  | 3=w24 | 6.0 (5.0 - 6.0) | p^13^ | **0.0013** | 0.0775 |  | 7.0 (6.0 - 9.0) | p^13^ | **<0.0001** | **<0.0001** |  |  |  |
|  | 4=w48 | 5.0 (5.0 - 6.0) | p^34^ | 1.0000 | 0.8434 |  | 7.0 (5.0 - 9.0) | p^34^ | **0.0127** | 0.0788 |  |  |  |
| **MMT-8**  **m. deltoideus**  (0-10) | 1=w0 | 8.0 (6.0 - 9.0) | p^12^ | **0.0005** | **0.0336** |  | 7.0 (5.0 - 8.0) | p^12^ | **<0.0001** | **0.0067** | **<0.0001** | **<0.0001** | |
|  | 2=w12 | 7.0 (6.0 - 8.0) | p^23^ | 0.0695 | 0.2492 |  | 7.0 (6.0 - 9.0) | p^23^ | **<0.0001** | **<0.0001** |  |  |  |
|  | 3=w24 | 7.0 (5.0 - 7.0) | p^13^ | **<0.0001** | **0.0025** |  | 9.0 (8.0 - 10.0) | p^13^ | **<0.0001** | **<0.0001** |  |  |  |
|  | 4=w48 | 6.0 (5.0 - 8.0) | p^34^ | 0.8145 | 0.2591 |  | 8.0 (7.0 - 9.0) | p^34^ | **0.0020** | 0.0534 |  |  |  |
| **MMT-8**  **m. biceps brachii**  (0-10) | 1=w0 | 9.0 (8.0 - 10.0) | p^12^ | **0.0007** | **0.0036** |  | 8.0 (6.0 - 9.0) | p^12^ | **<0.0001** | **0.0016** | **<0.0001** | **<0.0001** | |
|  | 2=w12 | 8.0 (7.0 - 10.0) | p^23^ | **0.0003** | **0.0153** |  | 8.0 (7.0 - 9.0) | p^23^ | **<0.0001** | **<0.0001** |  |  |  |
|  | 3=w24 | 7.0 (6.0 - 8.0) | p^13^ | **<0.0001** | **<0.0001** |  | 10.0 (9.0 - 10.0) | p^13^ | **<0.0001** | **<0.0001** |  |  |  |
|  | 4=w48 | 8.0 (6.0 - 9.0) | p^34^ | **0.0079** | **0.0117** |  | 9.0 (8.0 - 10.0) | p^34^ | **0.0002** | **0.0044** |  |  |  |
| **MMT-8**  **wrist extensors**  (0-10) | 1=w0 | 10.0 (10.0 - 10.0) | p^12^ | 0.5035 | 0.5338 |  | 9.0 (9.0 - 10.0) | p^12^ | **0.0026** | **0.0123** | **<0.0001** | 0.1220 | |
|  | 2=w12 | 10.0 (10.0 - 10.0) | p^23^ | 0.2660 | 0.2913 |  | 10.0 (9.0 - 10.0) | p^23^ | **0.0086** | 0.0548 |  |  |  |
|  | 3=w24 | 10.0 (9.0 - 10.0) | p^13^ | 0.1536 | 0.1697 |  | 10.0 (10.0 - 10.0) | p^13^ | **<0.0001** | **0.0002** |  |  |  |
|  | 4=w48 | 10.0 (9.0 - 10.0) | p^34^ | 0.7704 | 0.7778 |  | 10.0 (9.0 - 10.0) | p^34^ | **0.0295** | 0.2069 |  |  |  |
| **MMT-8**  **m. quadriceps femoris**  (0-10) | 1=w0 | 10.0 (9.0 - 10.0) | p^12^ | **0.0127** | 0.3014 |  | 10.0 (4.0 - 10.0) | p^12^ | **0.0078** | 0.0958 | **<0.0001** | 0.1648 | |
|  | 2=w12 | 9.0 (8.0 - 10.0) | p^23^ | 0.2353 | 0.3344 |  | 10.0 (7.0 - 10.0) | p^23^ | **0.0328** | 0.1608 |  |  |  |
|  | 3=w24 | 10.0 (6.0 - 10.0) | p^13^ | **0.0130** | 0.1464 |  | 10.0 (10.0 - 10.0) | p^13^ | **0.0133** | 0.0935 |  |  |  |
|  | 4=w48 | 9.0 (9.0 - 10.0) | p^34^ | 0.3972 | 0.0747 |  | 10.0 (9.0 - 10.0) | p^34^ | 0.1189 | 0.3883 |  |  |  |
| **MMT-8**  **m. gluteus maximus**  (0-10) | 1=w0 | 5.0 (3.0 - 8.0) | p^12^ | **0.0071** | 0.0759 |  | 5.0 (3.0 - 6.0) | p^12^ | **<0.0001** | **<0.0001** | **<0.0001** | **<0.0001** | |
|  | 2=w12 | 5.0 (3.0 - 6.0) | p^23^ | 0.1253 | 0.3781 |  | 6.0 (5.0 - 8.0) | p^23^ | **<0.0001** | **<0.0001** |  |  |  |
|  | 3=w24 | 4.0 (3.0 - 5.0) | p^13^ | **0.0026** | **0.0463** |  | 8.0 (7.0 - 10.0) | p^13^ | **<0.0001** | **<0.0001** |  |  |  |
|  | 4=w48 | 5.0 (3.0 - 6.0) | p^34^ | 0.1347 | **0.0097** |  | 7.0 (5.0 - 9.0) | p^34^ | **0.0022** | 0.0528 |  |  |  |
| **MMT-8**  **m. gluteus medius**  (0-10) | 1=w0 | 6.0 (4.0 - 9.0) | p^12^ | **0.0384** | 0.0754 |  | 7.0 (4.0 - 8.0) | p^12^ | **0.0003** | **0.0022** | **<0.0001** | **<0.0001** | |
|  | 2=w12 | 5.0 (3.0 - 8.0) | p^23^ | 0.2990 | 0.9026 |  | 7.0 (6.0 - 9.0) | p^23^ | **<0.0001** | **0.0002** |  |  |  |
|  | 3=w24 | 5.0 (4.0 - 7.0) | p^13^ | **0.0023** | 0.1272 |  | 9.0 (7.0 - 10.0) | p^13^ | **<0.0001** | **<0.0001** |  |  |  |
|  | 4=w48 | 6.0 (4.0 - 9.0) | p^34^ | **0.0290** | **0.0273** |  | 8.0 (6.0 - 9.0) | p^34^ | **0.0003** | **0.0050** |  |  |  |
| **MMT-8**  **ankle dorsiflexors**  (0-10) | 1=w0 | 10.0 (9.0 - 10.0) | p^12^ | **0.0216** | 0.1083 |  | 9.0 (7.0 - 10.0) | p^12^ | **0.0026** | **0.0422** |  |  | |
|  | 2=w12 | 10.0 (8.0 - 10.0) | p^23^ | 0.1836 | 0.1539 |  | 10.0 (8.0 - 10.0) | p^23^ | **0.0062** | **0.0142** |  |  | |
|  | 3=w24 | 9.0 (8.0 - 10.0) | p^13^ | **0.0050** | **0.0171** |  | 10.0 (10.0 - 10.0) | p^13^ | **0.0019** | **0.0054** | **<0.0001** | **0.0037** | |
|  | 4=w48 | 9.0 (9.0 - 10.0) | p^34^ | 0.3282 | 0.4164 |  | 10.0 (9.0 - 10.0) | p^34^ | **0.0238** | 0.1537 |  |  | |
| **MMT**  **m. triceps brachii**  (0-10) | 1=w0 | 9.0 (9.0 - 10.0) | p^12^ | **0.0357** | 0.1412 |  | 9.0 (7.0 - 9.0) | p^12^ | **<0.0001** | **0.0002** | **<0.0001** | **<0.0001** | |
|  | 2=w12 | 9.0 (8.0 - 10.0) | p^23^ | **0.0063** | **0.0339** |  | 10.0 (9.0 - 10.0) | p^23^ | **0.0223** | 0.0835 |  |  |  |
|  | 3=w24 | 8.0 (6.0 - 9.0) | p^13^ | **0.0008** | **0.0088** |  | 10.0 (9.0 - 10.0) | p^13^ | **<0.0001** | **0.0001** |  |  |  |
|  | 4=w48 | 9.0 (7.0 - 10.0) | p^34^ | 0.1424 | **0.0287** |  | 10.0 (8.0 - 10.0) | p^34^ | **0.0090** | 0.0761 |  |  |  |
| **MMT**  **m. iliopsoas**  (0-10) | 1=w0 | 5.0 (3.0 - 8.0) | p^12^ | **0.0030** | 0.0560 |  | 4.0 (3.0 - 5.0) | p^12^ | **<0.0001** | **<0.0001** | **<0.0001** | **<0.0001** | |
|  | 2=w12 | 5.0 (3.0 - 6.0) | p^23^ | 0.1073 | 0.4122 |  | 6.0 (5.0 - 7.0) | p^23^ | **<0.0001** | **<0.0001** |  |  |  |
|  | 3=w24 | 4.0 (3.0 - 5.0) | p^13^ | **0.0035** | **0.0307** |  | 8.0 (6.0 - 9.0) | p^13^ | **<0.0001** | **<0.0001** |  |  |  |
|  | 4=w48 | 5.0 (3.0 - 5.0) | p^34^ | 0.4791 | 0.5752 |  | 7.0 (5.0 - 8.0) | p^34^ | **0.0015** | **0.0459** |  |  |  |
|  |  |  |  |  |  |  |  |  |  |  |  |  | |
| **FIS total score**  (160-0) | 1=w0 | 60.0 (41.0 -76.0) | p^12^ | 0.6125 | 0.4027 |  | 41.0 (28.0 -59.0) | p^12^ | 0.1098 | 0.3996 | 0.3203 | 0.9556 | |
|  | 2=w12 | 53.0 (38.0 - 83.0) | p^23^ | 0.3439 | 0.4763 |  | 37.0 (27.0 -58.0) | p^23^ | 0.4807 | 0.3323 |  |  |  |
|  | 3=w24 | 60.0 (40.0 - 87.0) | p^13^ | 0.7235 | 0.8781 |  | 39.0 (12.0 -59.0) | p^13^ | 0.1012 | 0.9020 |  |  |  |
|  | 4=w48 | 55.0 (37.0 - 101.0) | p^34^ | 0.4771 | 0.8045 |  | 42.0 (9.0 -59.0) | p^34^ | 0.4549 | 0.8705 |  |  |  |
| **SF-36 PCS**  (16.6-57.9) | 1=w0 | 20.4 (13.8 - 34.0) | p^12^ | 0.5791 | 0.7317 |  | 30.9 (27.5 - 40.4) | p^12^ | 0.0778 | **0.0238** | 0.1428 | 0.1320 | |
|  | 2=w12 | 20.7 (16.4 - 26.3) | p^23^ | 0.9479 | 0.8756 |  | 33.6 (25.3 - 48.0) | p^23^ | 0.4209 | 0.5949 |  |  |  |
|  | 3=w24 | 20.1 (15.3 - 25.9) | p^13^ | 0.5533 | 0.5763 |  | 35.3 (27.1 - 48.4) | p^13^ | 0.0567 | **0.0467** |  |  |  |
|  | 4=w48 | 24.9 (15.3 - 32.7) | p^34^ | 0.2296 | 0.2991 |  | 33.6 (23.4 - 47.1) | p^34^ | 0.2264 | 0.9803 |  |  |  |
| **SF-36 MCS**  (5.5-63.6) | 1=w0 | 41.9 (31.4 - 54.0) | p^12^ | 0.9582 | 0.4567 |  | 45.3 (36.1 - 56.0) | p^12^ | 0.7824 | 0.6463 | 0.5446 | 0.4203 | |
|  | 2=w12 | 41.5 (32.6 - 52.0) | p^23^ | 0.3450 | 0.7309 |  | 46.7 (36.6 - 54.8) | p^23^ | 0.9417 | 0.2995 |  |  |  |
|  | 3=w24 | 45.1 (36.2 - 52.2) | p^13^ | 0.3117 | 0.3164 |  | 46.4 (37.0 - 57.0) | p^13^ | 0.9196 | 0.5297 |  |  |  |
|  | 4=w48 | 36.2 (31.1 - 50.1) | p^34^ | **0.0095** | 0.0696 |  | 44.7 (40.2 - 56.5) | p^34^ | 0.8498 | 0.5436 |  |  |  |
| **Acronyms:** IQR, inter-quartile range; MMT-8, Manual Muscle Testing of eight muscles; FIS, Fatigue Impact Scale; SF-36, Medical Outcomes Study 36-item Short Form Health Survey; PCS, SF-36 Physical Component Score; SF-36 MCS, SF-36 Mental Component Score; w0, week 0 (baseline); w12, week 12 (after 12 weeks of intervention); w24, week 24 (after 24 weeks of intervention); w48, week 48 (24 weeks after the end of intervention); p^12^ – difference between time 1 and 2; p^23^ – difference between time 2 and 3; p^13^ – difference between time 1 and 3; p^34^ – difference between time 3 and 4; Unadjusted data are presented as median (IQR). Statistically significant differences (p<0.05) are marked in bold. Intra-group comparisons were performed by one way ANOVA. Inter-group comparisons were performed by two way ANOVA. P-values are provided for both unadjusted data and for data adjusted for MMT-8, creatine kinase and the current prednisone equivalent dose. | | | | | | | | | | | | |  |

**Table S3. Perceived exertion and disease activity assessed by systemic levels of**

**markers for inflammation and muscle damage**

| **Parameter** | **Group** | **Week 0** | **Week 24** | **p-value** |
| --- | --- | --- | --- | --- |
| **Borg CR-10** | CG | 4.59 ±0.39 | 4.65 ± 0.42 | 0.4338 |
|  | IG | 4.08 ± 0.34 | 3.81 ± 0.27 | 0.5165 |
| **CRP**  **(mg/L)** | CG | 8.19 ± 3.99 | 11.84 ± 4.98 | 0.2316 |
|  | IG | 3.89 ± 0.72 | 4.50 ± 1.22 | 0.9153 |
| **ESR**  **(mm/h)** | CG | 20.9 ± 3.6 | 22.5 ± 3.9 | 0.7453 |
|  | IG | 17.7 ± 2.6 | 17.3 ± 2.3 | 0.8705 |
| **CK**  **(µkat/L)** | CG | 5.37 ± 2.63 | 3.23 ± 0.88 | 0.2129 |
|  | IG | 9.08 ± 2.52 | 7.51 ± 0.03 | 0.4517 |
| **LD**  **(µkat/L)** | CG | 4.45 ± 0.53 | 4.03 ± 0.23 | 0.8987 |
|  | IG | 4.42 ± 0.32 | 4.14 ± 0.28 | 0.8661 |
| **Myoglobin**  **(µg/L)** | CG | 236.2 ± 103.7 | 93.5 ± 76.2 | 0.1304 |
|  | IG | 261.8 ± 72.82 | 174.3 ± 41.92 | 0.4270 |
| **IL-1β**  **(pg/mL)** | CG | 1.32 ± 0.28 | 2.38 ± 0.34 | **0.0104** |
|  | IG | 1.25 ± 0.29 | 1.61 ± 0.26 | 0.0854 |
| **IL-6**  **(pg/mL)** | CG | 1.52 ± 0.41 | 2.61 ± 0.419 | 0.0674 |
|  | IG | 2.06 ± 1.23 | 2.18 ± 0.45 | 0.1963 |
| **IL-8**  **(pg/mL)** | CG | 9.64 ± 1.35 | 8.22 ± 0.81 | 0.2572 |
|  | IG | 5.89 ± 0.74 | 5.80 ± 0.40 | 0.4809 |
| **TNF**  **(pg/mL)** | CG | 58.42 ± 5.73 | 41.41 ± 2.12 | **<0.0001** |
|  | IG | 52.81 ± 6.99 | 36.66 ± 2.82 | **0.0013** |
| **MCP-1 (CCL2) (pg/mL)** | CG | 83.83 ± 18.22 | 42.40 ± 6.44 | **0.0043** |
|  | IG | 57.88 ± 7.10 | 45.46 ± 6.40 | 0.0796 |
| **Acronyms:** Data are presented as the mean ± standard error of the mean. Statistically significant differences (p<0.05) are marked in bold. Borg CR-10, Borg Category-Ratio 10 scale of perceived muscle exertion from 0 (nothing at all) to 10 (extremely strong, almost maximal); CRP, C-reactive protein; CK, creatine phosphokinase; LD, lactate dehydrogenase; IL, interleukin; TNF, tumor necrosis factor; MCP-1 (CCL2), monocyte chemoattractant protein-1, CG, control group; IG, intervention group. | | | | |
|  | | | | |

**Table S4. Relative mRNA expression of proinflammatory cytokines and chemokines in muscle biopsy prior and after the intervention (n=7)**

| **Parameter** | **Week 0** | **Week 24** | **p-value** |
| --- | --- | --- | --- |
| **IL-1β** | 1.0 ± 0.472 | 0.113 ± 0.036 | 0.1563 |
| **IL-6** | 1.0 ± 0.728 | 0.211 ± 0.067 | 0.4688 |
| **IL-8** | 1.0 ± 0.563 | 1.320 ± 0.839 | 1.0000 |
| **TNF** | 1.0 ± 0.256 | 0.169 ± 0.035 | **0.0012** |
| **MCP-1 (CCL2)** | 1.0 ± 0.404 | 0.220 ± 0.058 | 0.1649 |
| **Acronyms:** Data are presented as the mean ± standard error of the mean. Statistically significant differences (p<0.05) are marked in bold. IL, interleukin; TNF, tumor necrosis factor; MCP-1 (CCL2), monocyte chemoattractant protein-1 | | | |
|  | | | |
